# Xylan synthesized by *Irregular Xylem 14* (*IRX14*) maintains the structure of seed coat mucilage in *Arabidopsis*

**DOI:** 10.1093/jxb/erv510

**Published:** 2016-02-01

**Authors:** Ruibo Hu, Junling Li, Xiaoyu Wang, Xun Zhao, Xuanwen Yang, Qi Tang, Guo He, Gongke Zhou, Yingzhen Kong

**Affiliations:** ^1^Qingdao Engineering Research Center of Biomass Resources and Environment, Qingdao Institute of Bioenergy and Bioprocess Technology, Chinese Academy of Sciences, Qingdao 266101, P.R. China; ^2^Key laboratory of Tobacco Genetic Improvement and Biotechnology, Tobacco Research Institute of Chinese Academy of Agricultural Sciences, Qingdao 266101, P.R. China

**Keywords:** Arabidopsis, crystalline cellulose, *IRX14*, mucilage, xylan.

## Abstract

During differentiation, the Arabidopsis seed coat epidermal cells synthesize and secrete large quantities of pectinaceous mucilage into the apoplast, which is then released to encapsulate the seed upon imbibition. In this study, we showed that mutation in *Irregular Xylem 14* (*IRX14*) led to a mucilage cohesiveness defect due to a reduced xylan content. Expression of *IRX14* was detected specifically in the seed coat epidermal cells, reaching peak expression at 13 days post-anthesis (DPA) when the accumulation of mucilage polysaccharides has ceased. Sectioning of the *irx14-1* seed coat revealed no visible structural change in mucilage secretory cell morphology. Although the total amount of mucilage was comparable with the wild type (WT), the partition between water-soluble and adherent layers was significantly altered in *irx14-1*, with redistribution from the adherent layer to the water-soluble layer. The monosaccharide composition analysis revealed that xylose content was significantly reduced in *irx14-1* water-soluble and adherent mucilage compared with the WT. The macromolecular characteristics of the water-soluble mucilage were modified in *irx14-1* with a loss of the larger polymeric components. In accordance, glycome profiling and dot immunoblotting of seed mucilage using antibodies specific for rhamnogalacturonan I (RG I) and xylan confirmed the ultra-structural alterations in the *irx14-1* mucilage. Meanwhile, the crystalline cellulose content was reduced in the *irx14-1* mucilage. These results demonstrated that *IRX14* was required for the biosynthesis of seed mucilage xylan, which plays an essential role in maintaining mucilage architecture potentially through altering the crystallization and organization of cellulose.

## Highlight

Xylan synthesized by *IRX14* maintains seed coat mucilage structure potentially through altering the crystallization and organization of cellulose.

## Introduction

In myxospermous species such as *Arabidopsis thaliana*, seed coat mucilage secretory cells (MSCs) synthesize and deposit large amounts of pectinaceous mucilage in the apoplast (Western *et al.*, 2000; Windsor *et al.*, 2000). Following mucilage synthesis, a secondary cell wall is synthesized and deposited, forming a volcano-shaped columella in the centre of the cell (Western *et al.*, 2000; Windsor *et al.*, 2000). At seed maturity, the epidermal cells have the central columella connected to reinforced radial cell walls and surrounded by doughnut-shaped mucilage under a primary cell wall. Hydration of mature seeds leads to an almost instantaneous mucilage release forming a gelatinous capsule surrounding the seed (Western *et al.*, 2000; Windsor *et al.*, 2000). The MSCs of Arabidopsis have been presented as an ideal model system for the study of the biosynthesis and modification of cell wall components (Arsovski *et al.*, 2010; Haughn and Western, 2012; Western, 2012; Francoz *et al.*, 2015; Voiniciuc *et al.*, 2015b;).

Staining of Arabidopsis mucilage with the pectate staining dye ruthenium red revealed the presence of two distinct layers: an outer, water-soluble layer and an inner, adherent layer tightly attached to the seed surface (Western *et al.*, 2000; Windsor *et al.*, 2000). Both layers have been demonstrated to be composed primarily of unbranched rhamnogalacturonan I (RG I) (Western *et al.*, 2000; Windsor *et al.*, 2000; Macquet *et al.*, 2007). A minor amount of homogalacturonan (HG) is also present within the adherent mucilage (Willats *et al.*, 2001), and its degree of methyl esterification varies, with a higher degree of variation in the outer layer than in the inner layer (Macquet *et al.*, 2007).

Apart from pectin, immunochemical analysis revealed that a small amount of diffuse cellulose was also present within the mucilage. Cellulose was interspersed with rays extending from the tip of the columella at the seed surface across the inner layer (Western *et al.*, 2000; Windsor *et al.*, 2000; Macquet *et al.*, 2007; Stork *et al.*, 2010). The presence of cellulose in the inner layer has been proved to be essential for mucilage adherence (Harpaz-Saad *et al.*, 2011; Mendu *et al.*, 2011a; Sullivan *et al.*, 2011). For example, *CELLULOSE SYNTHASE5* (*CESA5*)/*MUM3* was shown to be responsible for the synthesis and proper deposition of cellulose in the inner adherent domain (Sullivan *et al.*, 2011). *cesa5*/*mum3* exhibited increased water-soluble mucilage accompanied by decreased adherent mucilage, suggesting a structural role of cellulose in anchoring the pectinaceous mucilage to the seed (Harpaz-Saad *et al.*, 2011; Mendu *et al.*, 2011a; Sullivan *et al.*, 2011). In addition, linkage analysis revealed small quantities of hemicellulose in the mucilage (Huang *et al.*, 2011; Walker *et al.*, 2011; Kong *et al.*, 2013; Yu *et al.*, 2014), which may cross-link with the cellulose in the mucilage similar to its proposed role in cell walls (Haughn and Western, 2012; Yu *et al.*, 2014; Voiniciuc *et al.*, 2015*a*, *b*). For example, glucomannans synthesized by CELLULOSE SYNTHASE-LIKE A2 (CSLA2) were proposed to modulate the structure of adherent mucilage, potentially through altering cellulose organization and crystallization (Yu *et al.*, 2014). Moreover, MUCI10 was responsible for the galactosylation of glucomannans synthesized by CSLA2 and affected mucilage architecture along with cellulosic rays (Voiniciuc *et al.*, 2015a).

Xylans are the major hemicellulose that cross-link with cellulose in the secondary walls of dicots and in both primary and secondary walls in commelinid monocots (Scheller and Ulvskov, 2010; Rennie and Scheller, 2014). Xylans are composed of a linear backbone of a β-1,4-linked d-xylose (Xyl) residues, which can be diversely substituted with arabinose (Ara), glucuronic acid (GlcA) and 4-*O*-methyl glucuronic acid (MeGlcA) depending on the species and tissue types (Ebringerová and Heinze, 2000). Based on the side chain substitutions, xylans are generally classified as glucuronoxylan (GX), the major hemicellulose in dicots, arabinoxylan (AX) and glucuronoarabinoxylan (GAX), the most abundant hemicelluloses in grasses (Ebringerová and Heinze, 2000).

Previous studies using reverse genetics approaches have revealed that several glycosyltransferases (GT) were involved in xylan biosynthesis in Arabidopsis. For instance, *IRX9*/*IRX9L* and *IRX14*/*IRX14L* from the GT43 family, and *IRX10*/*IRX10L* from the GT47 family are required for the elongation of the xylan backbone (Brown *et al.*, 2007, 2009; Wu *et al.*, 2009, 2010; Keppler and Showalter, 2010; Lee *et al.*, 2010). *IRX9*, *IRX14*, and *IRX10* have been proved to play major roles in xylan backbone biosynthesis, with their close homologues *IRX9L*, *IRX14L*, and *IRX10L* showing partially redundant roles (Wu *et al.*, 2009, 2010; Keppler and Showalter, 2010; Lee *et al.*, 2010). Loss of function of *IRX9*, *IRX14*, or *IRX10* resulted in decreased xylan synthase activity, reduced xylan content, and xylan with short backbones. Mutations for each of these gene pairs (*IRX9*/*IRX9L*, *IRX10*/*IRX10L*, or *IRX14*/*IRX14L*) significantly enhanced the phenotype of the single mutants (Wu *et al.*, 2009, 2010; Keppler and Showalter, 2010; Lee *et al.*, 2010). Interestingly, a recent study indicated that these three gene pairs play equally important roles rather than major and minor roles in xylan biosynthesis. *IRX9L*, *IRX10L*, and *IRX14* are mainly responsible for the synthesis of xylan in primary cell walls, whereas *IRX9*, *IRX10*, and *IRX14L* predominantly synthesize xylan in secondary cell walls (Mortimer *et al.*, 2015). In addition, *IRX7* (*FRA8*)/*IRX7L* (*F8H*) from the GT47 family, *IRX8* (*GAUT12*) and *PARVUS* from the GT8 family are responsible for the synthesis of the oligosaccharide at the reducing end of xylans (Brown *et al.*, 2007; Lee *et al.*, 2007, 2009; Pena *et al.*, 2007). Mutations in *IRX7*, *IRX8* or *PARVUS* led to a decreased xylan content and the lack of the reducing end oligosaccharide sequence, while the xylan backbone elongation activity was retained (Lee *et al.*, 2007, 2009; Pena *et al.*, 2007).

As a major hemicellulose component in cell walls, xylan biosynthesis has been extensively studied in secondary cell wall formation. However, no report on the function and biosynthesis of xylan in seed coat mucilage has been found to date. In this study, we showed that *IRX14* was essential for the biosynthesis of xylan in mucilage and played important roles in maintaining normal mucilage structure in the seed coat. Although *irx14-1* produced normal amounts of mucilage, the cohesive properties of the mucilage were significantly altered resulting from the fact that most of the pectinaceous components of the inner adherent mucilage layer were mal-distributed into the water-soluble layer. In addition, the macromolecular characteristics of the water-soluble mucilage in *irx14-1* were dramatically altered, and the crystalline cellulose content was significantly reduced in *irx14-1* mucilage. Our results demonstrate that xylan synthesized by *IRX14* functioned in maintaining the proper structure of mucilage potentially through its interaction with cellulose.

## Materials and methods

### Plant material and growth conditions

T-DNA insertion lines for *irx14-1* (SALK_038212), *irx14-2* (CS400996), *irx9-1* (SALK_058238), *irx14l* (SALK_066961), and *irx9l* (SALK_037323) were obtained from ABRC (http://www.arabidopsis.org). Homozygous lines were identified by genotyping using the primers indicated in Supplementary Table S1 at *JXB* online. Seeds were surface-sterilized and sown on to half-strength Murashige and Skoog (1/2 MS) medium, stratified in the dark for 2 d at 4 °C and germinated at 21 °C under a 16/8h day/night photoperiod. Ten-day-old plants were transferred to soil in growth chambers under the same conditions.

### Ruthenium red staining of seed mucilage

Seeds were imbibed for 2h in water with or without shaking, then stained in 0.01% (w/v) ruthenium red (Sigma-Aldrich) for 30min at room temperature. Following a brief rinse with de-ionized water, seeds were photographed with a BX51 light microscope (OLYMPUS).

### Microscopy and histology

For resin embedding and sectioning, developing seeds staged at 4, 7, 10, and 13 DPA were fixed in 2.5% (w/v) glutaraldehyde in 0.1M phosphate-buffered saline (PBS) (pH 7.0) overnight at 4 °C. After washing, samples were post-fixed for 1h in 1% (v/v) osmium tetraoxide in PBS, dehydrated through a gradient ethanol series and subsequently embedded in Spurr’s resin. Sections (1 μm) were cut and stained with 1% toluidine blue O dissolved in 0.1M PBS and washed with de-ionized water. Images were captured with a BX51 microscope (OLYMPUS).

For Scanning Electron Microscopy (SEM), mature dry seeds were mounted on stubs, coated with platinum in an E1045 ion sputter coater (Hitachi), and imaged using a S4800 scanning electron microscopy (Hitachi) with an accelerating voltage of 20kV.

For polarized light microscopy, seeds were imbibed in water for 30min before being mounted on a glass slide. Seed birefringence was observed with a Nikon Eclipse E600 POL microscope.

### Mucilage extraction

Three independent samples of 100mg seeds were extracted sequentially with water and 2M NaOH (with 3mg ml^–1^ NaBH_4_) for 1h each with shaking on an orbital shaker at room temperature (Western *et al.*, 2001). The resulting suspension was centrifuged to pool supernatants, and 2M NaOH extracts were neutralized with acetic acid, then dialysed extensively against running water for 36h before being freeze-dried.

### Determination of monosaccharide composition

Two mg of water-soluble and adherent mucilage was hydrolysed with 1ml of 2M trifluoroacetic acid (TFA) for 2h at 121 °C. The TFA was evaporated under a stream of nitrogen. The hydrolysates were derivatized with 1-phenyl-3-methyl-5-pyrazolone and 0.3M NaOH at 70 °C for 30min, extracted with chloroform three times and then analysed on a Hypersil ODS-2 C18 column (4.6×250mm; Thermo Scientific) using a Waters HPLC System. The derivatives were eluted with 82% (v/v) PBS (0.1M, pH 7.0) and 18% (v/v) acetonitrile at 1ml min^−1^, and monitored at 245nm. Neutral sugar standards (fucose, arabinose, rhamnose, galactose, glucose, mannose, and xylose) and an acid sugar standard (galacturonic acid) were included in the analysis.

### FTIR spectroscopy

Water- and 2M NaOH-extracted mucilage was used for FTIR analysis. Samples were prepared by dissolving 2mg mucilage with 20mg KBr in 50 μl water. The mixture was incubated overnight at 50 °C. Pellets were made by mixing 2mg mucilage-KBr mixture with 23mg KBr, and pressed into 7mm films. Spectra were collected using a Thermo Nicolet Nexus 470 spectrometer (Thermo Scientific) over the range of 4 000-800cm^−1^. For each spectrum, 200 scans were performed at 8cm^−1^ resolution. Subtraction plots between WT and *irx14-1* were generated using OMNIC software (Thermo Nicolet) and spectral differences were cross-referenced to identify peaks linked to pectin, xylan, and cellulose (Kacurácová *et al.*, 2000). Only the spectra between 1 900 and 800cm^−1^ were used for analysis.

### Macromolecular characterization

High-performance size-exclusion chromatography (HP-SEC) was performed on a system comprising of tandemly connected Shodex OHpak SB-802 and SB-806 columns, coupled with a differential refractive index detector (Wyatt Optilab rEX). Elution was carried out with 50mM NaNO_3_ at a flow rate of 1ml min^−1^. Samples were solubilized in 50mM NaNO_3_ and filtered through disposable syringe filters (0.22 μm, Millipore) before injection. Samples were injected automatically through a 0.5ml loop in an auto-sampler. Data analysis was performed using Astra software.

For electrophoresis of mucilage polymers, extracted water-soluble and adherent mucilage was re-suspended in water at a concentration of 0.5mg ml^−1^, and 200 μl was loaded in each lane. Electrophoresis was carried out in a 0.7% agarose gel prepared in TAE buffer at 60V (6V cm^−1^) for 2.5h. After electrophoresis, the gel was stained in 0.01% (w/v) ruthenium red (Sigma-Aldrich) solution for 30min and then photographed.

### Glycosyl linkage analysis

Total mucilage was extracted by vigorously shaking in 50mM EDTA using a Retsch MM300 TissueLyser (Qiagen). The reduction of the uronic acids to their respective 6,6-dideuterio derivatives was carried out as described by Gibeaut and Carpita (1991). After reduction, mucilage samples were extensively dialysed against running water and lyophilized, then solubilized in 200 µl anhydrous DMSO. Methylation was performed as described by Yu et al.( 2014). The methylated polymers purified by chloroform extraction were hydrolysed in 2M TFA. The sugars were then reduced with NaBD_4_ and acetylated. Partially methylated alditol acetates were dissolved in methylene chloride and analysed by GC–MS as described previously (Yu *et al.*, 2014).

### Seed cell wall preparation

Whole dry seeds and seeds lacking mucilage were frozen in liquid nitrogen and ground to a fine powder using a Retsch MM300 TissueLyzer (Qiagen, Germany). Ten mg of ground seeds were de-starched with a total starch assay kit (Megazyme) according to the manufacturer’s protocol, then washed twice with 2ml of 80% (v/v) ethanol, and centrifuged at 5 000rpm for 10min. The ethanol was removed and the pellet was washed sequentially with 95% (v/v) ethanol, 100% ethanol, and twice with 100% acetone. After washing, the samples were dried under a vacuum at 60 °C to obtain the alcohol-insoluble residue (AIR).

### Crystalline cellulose quantification

For the determination of the crystalline cellulose content, 20mg AIR was hydrolysed in 2ml of 2M TFA at 121°C for 2h. After centrifugation, the pellets were suspended in 2ml of Updegraff reagent [acetic acid:nitric acid:water, 8:1:2 by vol. ] (Updegraff, 1969), and boiled at 100 °C for 1h. The crystalline cellulose pellets were collected by centrifugation at 10 000rpm for 10min, and hydrolysed in 3ml of 72% (w/v) H_2_SO_4_ for 30min. The amounts of crystalline cellulose were quantified colorimetrically using the anthrone assay (Laurentin and Edwards, 2003).

### Immunolabelling of mucilage

Whole seed immunolabelling was performed with two xylan-specific antibodies CCRC-M139 (Pattathil *et al.*, 2010) and LM11 (McCartney *et al.*, 2005), and the carbohydrate-binding module CBM3a that binds to crystalline cellulose (Dagel *et al.*, 2011). Whole seeds were first blocked with PBS containing 3% (w/v) fat-free milk powder (MP/PBS) for 30min, and then incubated in 10-fold diluted primary antibodies in MP/PBS for 1.5h. Seeds were then washed with PBS and incubated with a 200-fold dilution of AlexaFluor488-tagged goat anti-mouse (CCRC-M139) or anti-rat (LM11) IgG in MP/PBS in darkness for 1h. Alternatively, seeds were incubated in MP/PBS containing 10mg ml^−1^ CBM3a for 1.5h, washed in PBS, and incubated in a 100-fold dilution of mouse anti-His monoclonal antibody for 1h. After washing with PBS, seeds were incubated in 200-fold diluted AlexaFluor488-tagged goat anti-mouse IgG in MP/PBS in darkness for 1h. After incubation, seeds were washed in PBS and counterstained for 5min with Calcofluor White (Sigma-Aldrich). Images were captured with a FluoView FV1000 confocal laser scanning microscope (OLYMPUS) using 405nm and 488nm lasers.

### Dot immunoblotting assays

Water and 2M NaOH mucilage extracts were re-suspended in water. A series of dilutions were prepared and a 1 μl aliquot was spotted on to a nitrocellulose membrane (Merck Millipore). After being air-dried, the membrane was blocked for 1h in MP/PBS, then incubated for 1.5h in a 10-fold dilution of primary antibodies. After washing three times with PBS, membranes were incubated for 1.5h in horseradish peroxidase (HRP)-conjugated anti-rat (for LM series) or anti-mouse (for CCRC series) secondary antibodies in a 1000-fold dilution in MP/PBS. Membranes were washed prior to colour development in substrate solution [25ml de-ionized water, 5ml methanol containing 10mg ml^−1^ 4-chloro-1-naphthol and 30 μl 6% (v/v) H_2_O_2_]. After incubation for 30min at room temperature, the blots were rinsed with de-ionized water and photographed.

### ELISA

Mucilage extractions (100 μg ml^−1^) in PBS were coated overnight at 4 °C on to 96-well microtitre plates (Costa 3599). Coating solutions were removed and 200 μl MP/PBS was added to each well. After blocking overnight at 4 °C, plates were washed and 100 μl primary antibodies at a 1/20 dilution in MP/PBS were added to each well. After incubation at 37 °C for 2h, plates were washed with PBS and incubated with HRP-conjugated anti-rat (JIM and LM series) or anti-mouse (CCRC series) secondary antibodies at a 1 000-fold dilution in MP/PBS for 2h. After washing with PBS, the antibody binding was determined by the addition of 150 μl HRP-substrate [12ml 0.1M sodium acetate buffer (pH 5.5), 200 μl tetramethylbenzidine, and 18 μl 6% (v/v) H_2_O_2_] to each well. The reaction was terminated after 5min by the addition of 50 μl of 2M H_2_SO_4_. The absorbance was measured at 450nm using a Synergy HT Multi-detection Microplate reader (Bio-Tek).

### Quantitative Real-Time RT-PCR (qRT-PCR) analysis

Total RNA was isolated from developing siliques using the CTAB method. Contaminating DNA was removed using RQ1 RNase-Free DNase I (Promega), and first-strand cDNA was synthesized with SuperScript III reverse transcriptase (Invitrogen) according to the manufacturer’s instructions. qRT-PCR was performed using SYBR Premix Ex Taq (TaKaRa) in the LightCycler 480 detection system (Roche). The analysis was performed using three technical replicates. Relative expression was calculated by the 2^−∆∆Ct^ method (Livak and Schmittgen, 2001) utilizing *ACTIN5* as the internal control. Gene-specific and reference gene primers are listed in Supplementary Table S1.

### *In situ* hybridization

The paraffin-embedded developing seed sections were used for *in situ* hybridization. Briefly, siliques were fixed in 4% paraformaldehyde in 0.1M PBS (pH 7.0) overnight at 4 °C. Samples were rinsed with PBS, then dehydrated through an escalating ethanol gradient (30, 50, 70, 80, 90, and 100%) for 15min each and subsequently embedded in paraffin (Sigma-Aldrich). Serial sections of 8 μm were cut, mounted on Superfrost Plus slides (Fisher), and dried overnight at 37 °C. The paraffin was dissolved from sections using xylene followed by rehydration, prehybridization, hybridization, and washes. To generate digoxygenin (DIG)-labelled probes, a specific fragment of *IRX14* (176bp) was amplified by PCR and cloned into the pGM-T vector (TIANGEN). Sense and antisense RNA probes were prepared using an *in vitro* transcription kit (Roche). Sections were viewed using a BX51 light microscope (OLYMPUS). Primer sequences for the probe preparation are listed in Supplementary Table S1.

## Results

### *IRX14* is preferentially expressed in the seed coat

To identify novel genes that control seed mucilage biosynthesis and modification, we re-analysed the publicly available microarray datasets for 42 Laser Capture Microdissected (LCM) seed samples across six developmental stages (GSE12404) (Le *et al.*, 2010). In this endeavour, more than 100 genes that were significantly up- or down-regulated during the six seed coat development stages were identified (Supplementary Fig. S1). Not surprisingly, almost all the previously reported genes involved in mucilage biosynthesis and modification were included in the list, proving the reliability of our analysis. Apart from these reported genes, we found that several more genes involved in xylan biosynthesis (e.g. *IRX14*, *IRX14L*, *IRX9*, and *IRX9L,* etc) were significantly up-regulated during seed coat development, suggesting a potential role of these genes in mucilage xylan biosynthesis. We ordered the T-DNA insertion mutants of these identified genes. By screening for altered ruthenium red-staining patterns of mucilage, genes including *IRX14* that are required for mucilage biosynthesis and/or modification were identified. This study only describes *IRX14* and other mutants will be reported elsewhere.

**Fig. 1.**
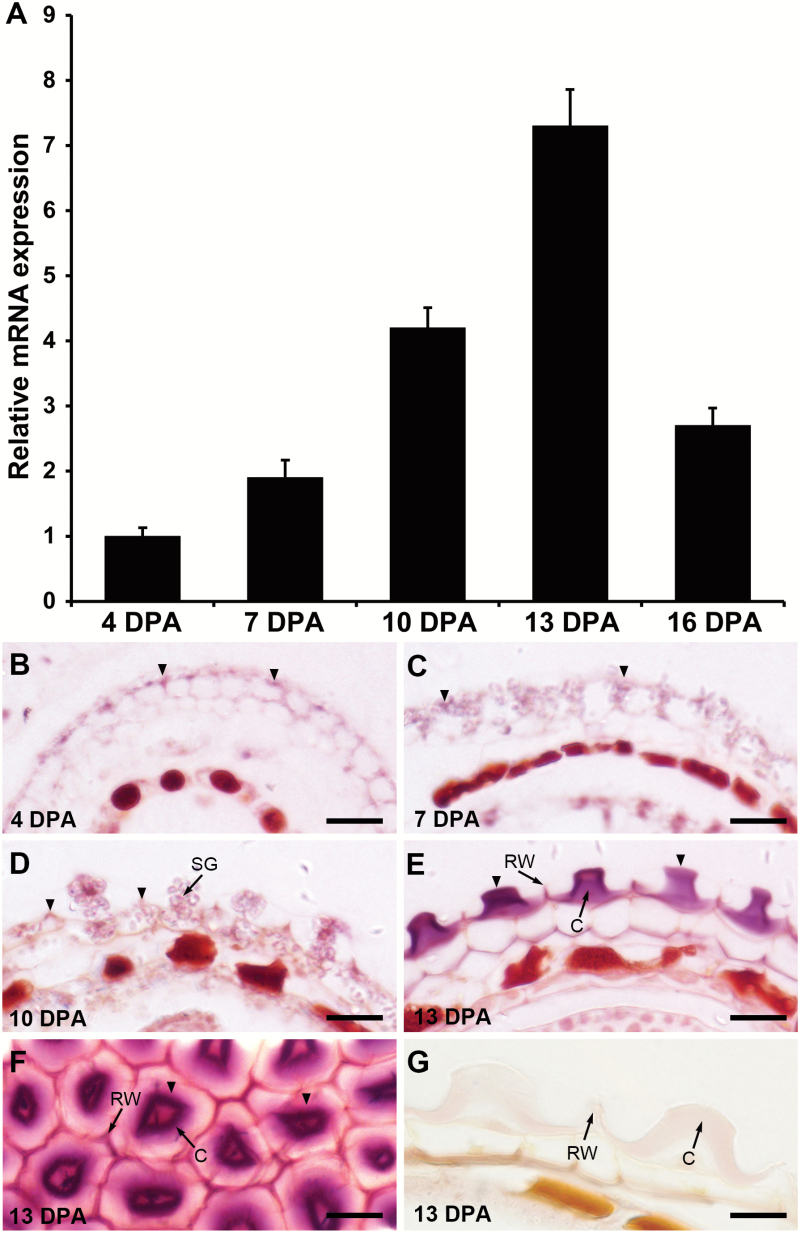
Expression of *IRX14* in developing seeds. (A) qRT-PCR analysis of *IRX14* expression during seed coat development staged at 4, 7, 10, 13, and 16 DPA. Error bars represent SD (*n*=3). The data are presented as relative fold change, where the *IRX14* expression level at 4 DPA is set at 1.0. (B–F) *In situ* hybridization using an *IRX14*-specific antisense probe. (G) *In situ* hybridization using an *IRX14*-specific sense probe in the seed coat of developing seeds. Arrowheads indicate the hybridization signals. C, Columella; RW, radial cell wall; SG, starch granule. Bar=20 μm.

We first validated the expression pattern of *IRX14* by quantitative real-time RT-PCR (qRT-PCR) in siliques at different developmental stages ranging from 4 days post-anthesis (DPA) to 16 DPA. *IRX14* expression increased gradually from the globular embryo stage (4 DPA) to the bending cotyledon stage (10 DPA), peaked at the mature green embryo stage (13 DPA), and then decreased at 16 DPA (Fig. 1A). At 13 DPA, mucilage synthesis was complete and structural modification may occur. To determine the precise spatial expression of *IRX14* in specific seed coat cells, *in situ* hybridization was performed using *IRX14*-specific probes. The hybridization signals were mainly detected in the epidermal cells of the outer integument where large quantities of mucilage was synthesized (Fig. 1B, D). Intense hybridization signals were detected in the central columella at 13 DPA (Fig. 1E, F). By contrast, no hybridization signal was detectable using the sense probe (Fig. 1G). In Arabidopsis, *IRX14* and its close homologue *IRX14L*, together with *IRX9* and *IRX9L*, are members of the GT43 family, which have been shown to act redundantly in xylan biosynthesis (Lee *et al.*, 2010; Wu *et al.*, 2010). The expression pattern of these GT43 family genes during seed development was examined using the LCM seed microarray datasets via the Bio-Array Resource eFP browser (Winter *et al.*, 2007). All these four genes exhibited seed coat-specific expression during seed development (Supplementary Fig. S2). The expression of *IRX9* was preferentially expressed at the bending cotyledon and mature green stage, which is similar to that of *IRX14,* while *IRX9L* and *IRX14L* were expressed in seed coats throughout the course of seed development, albeit with no large fluctuations. The seed coat-specific expression patterns of *IRX14* and its homologues prompted us to investigate their potential roles in mucilage biosynthesis and/or modification further.

### The mucilage layer is thinner in *irx14* mutants compared with the WT

**Fig. 2.**
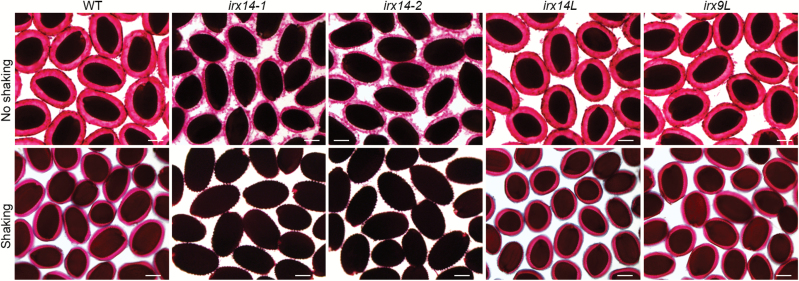
*IRX14* is involved in maintaining the adherent seed mucilage structure. Staining of seed mucilage by ruthenium red. WT, *irx14-1*, *irx14-2*, *irx14l*, and *irx9l* seeds were stained without shaking or after imbibition and shaking for 2h in water. Bar=100 μm.

To investigate the role of *IRX14* and its homologues in seed coat mucilage, we isolated *irx14*, *irx14l*, *irx9*, and *irx9l* homozygous insertion mutants. Ruthenium red staining of homozygous seeds showed that two *irx14* mutants (*irx14-1* and *irx14-2*) exhibited significant mucilage defects with a thinner mucilage halo compared with the WT (Fig. 2). Direct ruthenium red staining of the *irx14* (*irx14-1* and *irx14-2*) seeds without shaking showed a much thinner layer of adherent mucilage compared with the WT. When stained with ruthenium red after shaking in water, only a tiny amount of the inner mucilage layer remained attached to the *irx14* seeds. By contrast, both *irx9l* and *irx14l* exhibited similar staining patterns of mucilage to the WT (Fig. 2). Unfortunately, the mucilage phenotype was not obtained for *irx9* seeds because the growth of the *irx9-1* mutant allele we used was severely retarded and no seeds were available under our growth conditions. In addition, the seed mucilage phenotypes of the *irx14 irx14l* double mutant could not be obtained due to its severely retarded growth, dramatically dwarfed plant stature, and severe sterility. Since two mutant alleles of *irx14* have similar mucilage defect phenotype, we used *irx14-1* for further analysis in subsequent experiments.

### Seed coat structure and mucilage biosynthesis are unaffected in *irx14-1*

To determine whether mutation in *IRX14* affects mucilage biosynthesis or seed coat morphology during seed coat development and differentiation, seed coat epidermal cells from *irx14-1* and the WT staged from 4–13 DAP were sequentially sectioned, stained with toluidine blue O, and examined by light microscopy. As shown in Supplementary Fig. S3, no obvious differences were observed in the timing or process of differentiation between *irx14-1* and WT seeds. Mucilage production in *irx14-1* seed coat epidermal cells appeared similar to that of the WT. Furthermore, cytoplasmic rearrangement and columella formation also occurred normally in the mutant epidermal cells. The structure of *irx14-1* seed coat epidermal cells was examined in more detail by Scanning Electron Microscopy (SEM) on mature dry seeds. The epidermal cells of WT and *irx14-1* seeds exhibited the characteristic polygonal shapes with a central columella and were equivalent in size, indicating no significant changes in the outer and radial cell walls in *irx14-1* seeds compared with the WT. These results indicate that seed coat structure and mucilage biosynthesis were unaffected in *irx14-1*.

### Water-soluble mucilage is increased in *irx14* seeds

**Fig. 3.**
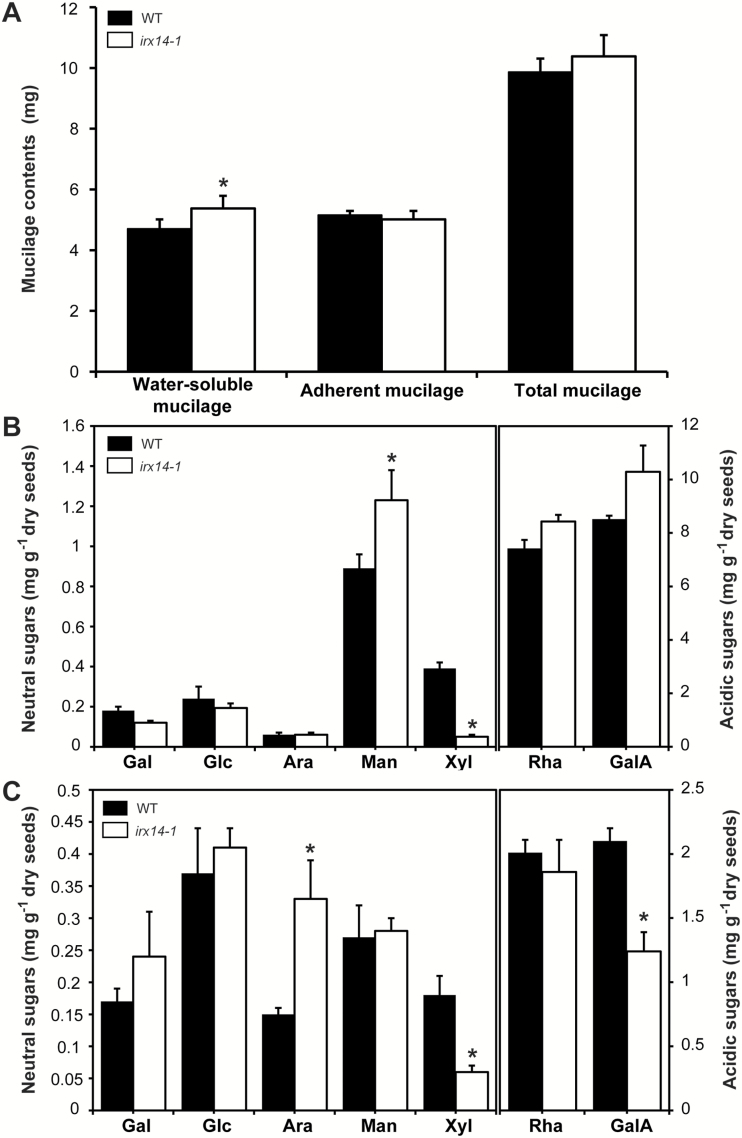
Mucilage weights and monosaccharide composition of *irx14-1* and the WT. (A) Mucilage weights from *irx14-1* and the WT. Water-soluble and adherent mucilage were sequentially extracted with water and 2M NaOH. Error bars indicate SD (*n*=3). (B, C) Monosaccharide composition of water-soluble and adherent mucilage from *irx14-1* and the WT, respectively. Results are given as the average (mg g^−1^ seeds) of triplicate assays ±SD. Asterisks indicated significant differences from WT (*P* <0.01).

To determine the changes in the water-soluble and adherent mucilage between *irx14-1* and the WT, seed mucilage was sequentially extracted with water and 2M NaOH. As shown in Fig. 3A, an increase of 14% in the amount of water-soluble mucilage was observed for *irx14-1* compared with the WT. Reciprocally, the amount of adherent mucilage was decreased by 3% in *irx14-1* compared with the WT. Nevertheless, there was no significant difference for the total amount of mucilage between *irx14-1* (10.38±0.52) and the WT (9.87±0.44). These results indicated that the *IRX14* mutation led to an increase in the level of water-soluble mucilage with a concomitant decrease in the adherent mucilage. The mal-distribution of mucilage between the outer and inner layers in *irx14-1* implies possible alterations of mucilage composition and structure.

### Xylose content is reduced in *irx14-1* mucilage

Considering the differences in mucilage extraction profiles, we further quantified the monosaccharide composition of *irx14-1* and WT mucilage using HPLC. A significant decrease (67–87%) was observed for Xyl content in both water-soluble and adherent mucilage in *irx14-1* (Fig. 3B, C). Meanwhile, mannose (Man) and Ara contents were significantly increased in the water-soluble and adherent mucilage of *irx14-1*, respectively. The increased levels of Ara and Man may result from the balancing effect among different hemicellulose components to counteract the decrease in xylose content. Furthermore, an up to 20% increase in total sugars was observed in *irx14-1* water-soluble mucilage, which was mainly due to the elevated levels in rhamnose (Rha) and galacturonic acid (GalA), indicating that *irx14-1* contained more water-soluble mucilage than the WT. By contrast, total sugars in adherent mucilage decreased by approximately 15% in *irx14-1*, also mainly due to reductions in Rha and GalA contents. This further confirms that the increased levels of sugars in water-soluble mucilage are caused by the re-distribution of some mucilage from the adherent layer to the water-soluble layer in *irx14-1*.

### FTIR reveals altered polysaccharide structures in *irx14-1* mucilage

Fourier Transform Infrared Spectroscopy (FTIR) has been proved to be an informative diagnostic tool in detecting subtle changes in cell wall carbohydrate structures based on signature peaks (Kacurácová *et al.*, 2000; Dean *et al.*, 2007; Mendu *et al.*, 2011b). To reveal the differences of composition between *irx14-1* and WT mucilage, we generated FTIR spectra for *irx14-1* and WT mucilage and subtraction spectra representing proportional differences between them (Supplementary Fig. S4). Within the FTIR subtraction plot, different peaks in RG I (1 017, 1 122, 1 150, 1 423, and 1 621cm ^−1^), xylan (978cm^−1^) and cellulose (898 and 1 034cm^−1^) were identified between WT and *irx14-1* spectra. These results corroborate the above monosaccharide composition analysis and further support that the composition of mucilage was altered in *irx14-1*.

### Macromolecular characteristics are altered in *irx14-1* soluble mucilage

**Fig. 4.**
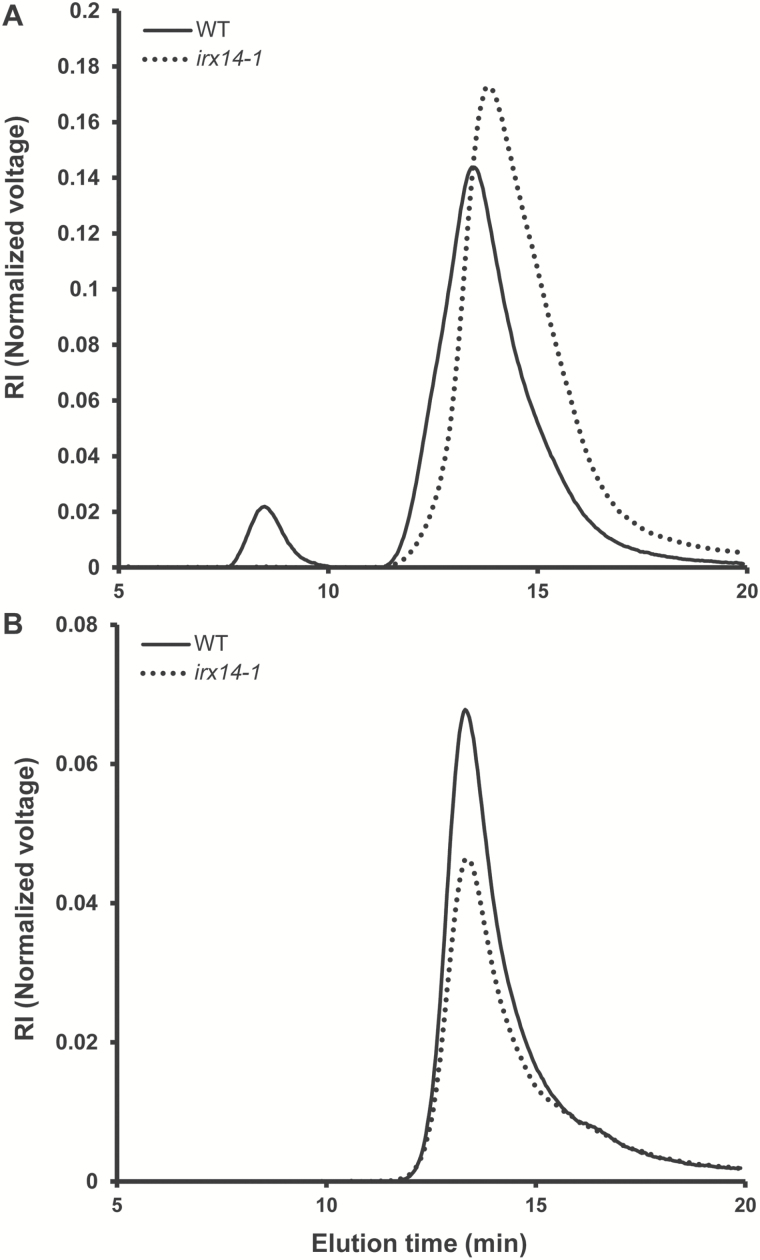
Macromolecular properties of water-soluble mucilage in *irx14-1* is altered. The mucilage extracted with water and 2M NaOH from *irx14-1* and WT seeds were separated by size-exclusion chromatography combined with refractive index detection. (A) Comparison of the elution profile of water-soluble mucilage in *irx14-1* and the WT. (B) Elution profile of adherent mucilage extracted by 2M NaOH in *irx14-1* and WT seeds. RI, Normalized refractive index signal voltage.

To explore whether the *irx14-1* mucilage phenotype is caused by a change in the size of the mucilage polysaccharides, high-performance size-exclusion chromatography (HP-SEC) combined with a refractive index detector and a multi-angle light-scattering detection was used to determine the macromolecular parameters of *irx14-1* and WT mucilage. Wild type water-soluble mucilage had an elution profile that separated two polymeric populations with different molar mass (Fig. 4). The first polymeric population was of an extremely high average molar mass (Mn approximately 30 000kDa), while the second one was of lower average molar mass (Mn approximately 750kDa). However, the macromolecular characteristics of the *irx14-1* water-soluble mucilage exhibited major differences from that of the WT. The higher molar mass population was totally absent in *irx14-1* water-soluble mucilage, and the second population eluted slightly later compared with the WT. By contrast, no significant difference for macromolecular characteristics was observed in 2M NaOH extracted mucilage between *irx14-1* and the WT.

Furthermore, we used gel electrophoresis to discern possible changes in size and/or charge in *irx14-1* mucilage. Although no significant difference was observed for adherent mucilage between *irx14-1* and the WT, the water-soluble mucilage displayed distinct staining patterns (Supplementary Fig. S5). The WT mucilage was distributed in a large smear in the gel lane, with much of the mucilage still remaining in the loading well. By contrast, the mucilage from *irx14-1* was mostly aggregated into a band with no material remaining in the loading well. These results indicate that the macromolecular characteristics of the water-soluble mucilage is altered in *irx14-1*.

### *IRX14* is essential for xylan synthesis in mucilage

To determine if subtle changes in the chemical structure of mucilage are present in *irx14-1*, we analysed the glycosyl linkage composition of total mucilage extracts (Table 1). Similar to the monosaccharide composition analysis (Fig. 3B, C), most of the composition of polysaccharides in *irx14-1* was comparable with that of WT. Consistent with previous studies (Huang *et al.*, 2011; Walker *et al.*, 2011; Kong *et al.*, 2013; Yu *et al.*, 2014), the mucilage in *irx14-1* and the WT was primarily composed of 2-Rha and 4-GalA, representing a relatively unbranched RG I backbone. The degree of branching of the RG I, as determined by the ratio of 2,4-Rha to 2-Rha, remained almost constant in *irx14-1* and the WT. An obvious change observed was a dramatic decrease in the xylan content in *irx14-1* compared with the WT (Table 1). Lower xylan content in *irx14-1* mucilage resulted from the severe reduction of 4-Xyl and t-Xyl. This indicated that *IRX14* is essential for the synthesis of the xylan backbone in mucilage.

**Table 1.** Sugar linkage composition of total mucilage extracted from *irx14-1* and WT seeds Total mucilage was extracted by vigorous shaking in 50mM EDTA. The results are given as the mean molar percentage of two independent assays, with variance less than 5% for all linkage groups.

Sugar and linkage	WT	*irx14-1*
Rhamnose		
t-Rha*p*	2.10	2.26
2-Rha*p*	33.25	35.78
2,3-Rha*p*	2.69	2.23
2,4-Rha*p*	6.01	4.97
Arabinose		
t-Ara*f*	0.27	0.24
3-Ara*f*	0.62	0.69
5-Ara*f*	0.50	0.51
2,5-Ara*f*	0.34	0.43
Xylose		
t-Xyl*p*	0.46	0.04
4-Xyl*p*	1.97	0.21
2,4-Xyl*p*	0.28	0.19
3,4-Xyl*p*	0.21	0.09
Mannose		
t-Man*p*	0.72	0.55
4-Man*p*	2.54	2.74
4,6-Man*p*	1.73	2.76
Galactose		
t-Gal*p*	0.59	0.62
3-Gal*p*	0.22	0.17
4-Gal*p*	0.16	0.14
6-Gal*p*	0.08	0.09
3,4-Gal*p*	0.16	0.30
3,6-Gal*p*	0.30	0.13
Glucose		
t-Glc*p*	0.22	0.14
4-Glc*p*	1.37	1.14
2,4-Glc*p*	0.24	0.24
4,6-Glc*p*	0.59	0.48
Galacturonic acid		
t-GalA*p*	5.55	5.47
4-GalA*p*	35.23	34.01
3,4-GalA*p*	1.59	3.39

### Crystalline cellulose is reduced in *irx14-1* mucilage

**Fig. 5.**
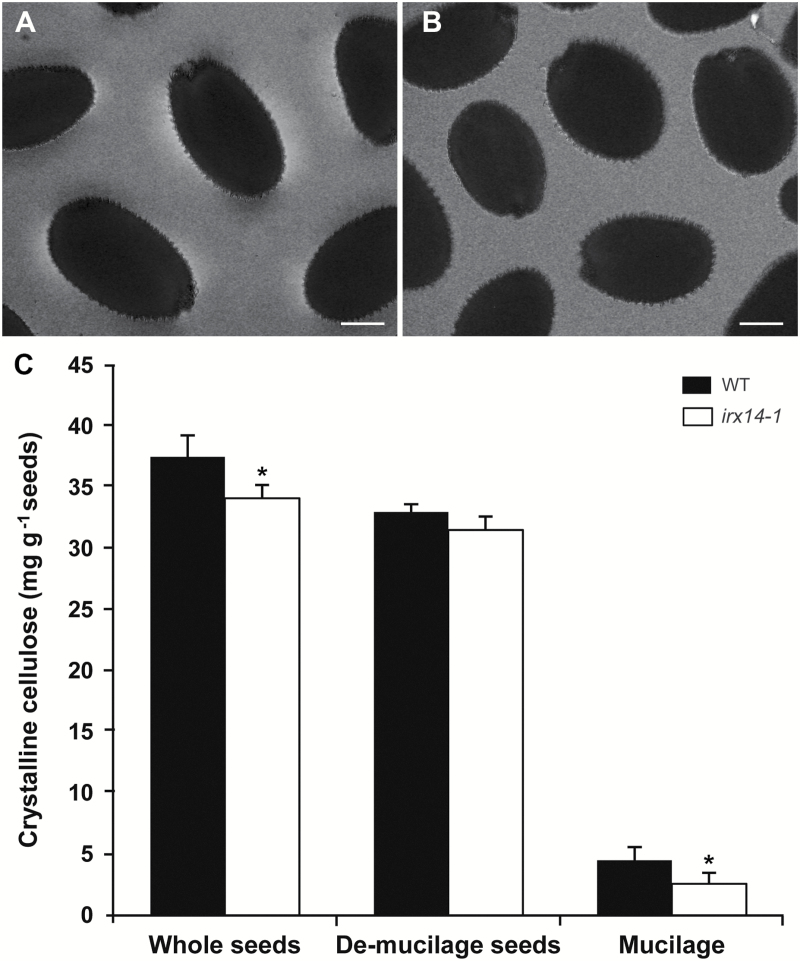
Crystalline cellulose content is reduced in *irx14-1*. (A, B) Visualization of polarized light birefringence by crystalline cellulose in adherent mucilage released from imbibed WT (A) and *irx14-1* (B) seeds. Bar=150 μm. (C) Quantification of crystalline cellulose contents in whole seeds, de-mucilaged seeds, and in the mucilage of *irx14-1* and the WT. Error bars represent SD (*n*=3). Asterisks indicate significant differences from the WT (*P* <0.05).

Previous studies indicated that cellulose plays an important role in maintaining mucilage adherence (Harpaz-Saad *et al.*, 2011; Mendu *et al.*, 2011a; Sullivan *et al.*, 2011). To determine the change in cellulose content in *irx14-1* mucilage, *irx14-1* and WT seeds imbibed in water were observed for birefringence produced by crystalline cellulose under polarized light (Fig. 5A, B). The outer epidermal cells of WT seeds exhibited strong birefringence with visible rays of crystalline cellulose within the adherent mucilage. By contrast, *irx14-1* displayed much less birefringence under polarized light, indicating that the crystalline cellulose content was dramatically reduced in *irx14-1* mucilage. Furthermore, quantification of crystalline cellulose contents in mucilage, de-mucilaged seeds, and whole seeds revealed that *irx14-1* mucilage had a 20% reduction in crystalline cellulose compared with the WT (Fig. 5C).

### The structure of mucilage is altered in *irx14-1*

**Fig. 6.**
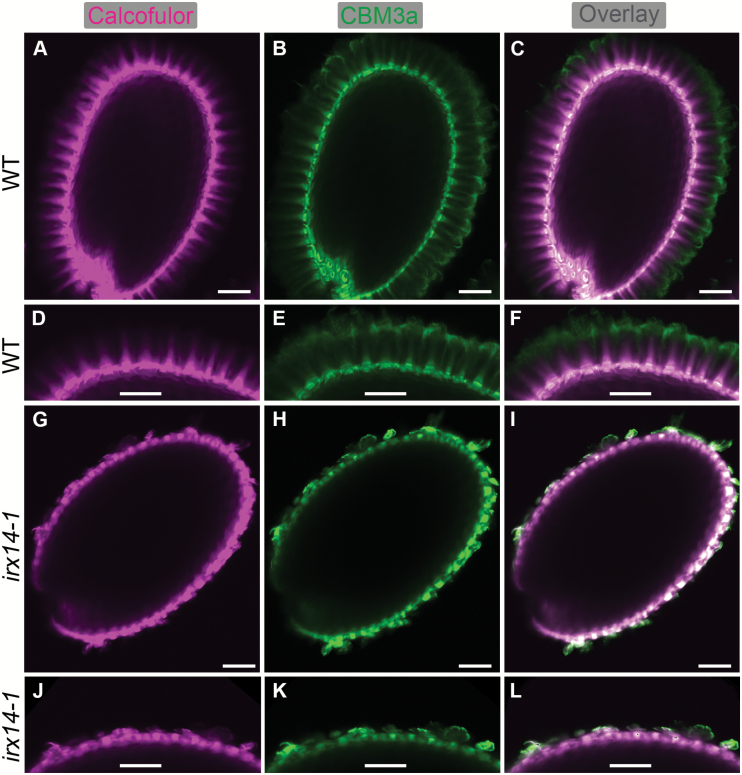
*In situ* labelling of crystalline cellulose in adherent mucilage from *irx14-1* and WT seeds. (A, D, G, J) Staining of β-glycans with Calcofluor White. (B, E, H, K) Indirect immunofluorescence detection of His-tagged CBM3a binding to crystalline cellulose. (C, F, I, L) Composite images of double labelling with Calcofluor and CBM3a. (D–F) and (J–L) correspond to the magnification of regions in (A–C) and (G–I), respectively. Bar=100 μm.

To examine further the structure and composition of mucilage in *irx14-1*, we performed whole mount immunolabelling on mature dry seeds. The carbohydrate-binding module CBM3a, which binds to crystalline cellulose (Dagel *et al.*, 2011), in parallel with Calcofluor, a fluorescent probe for β-glycans, was used to examine the spatial distribution of cellulose in the mucilage (Fig. 6). Calcofluor labelling of WT mucilage revealed intense rays radiating from the tops of the columella and across the inner layer of the seed mucilage, whereas labelling of Calcofulor was only detectable in mucilage around the columella in *irx14-1*, and the diffuse staining of rays was almost completely absent. CBM3a displayed a similar staining pattern to that of Calcofluor in WT adherent mucilage, with intense labelling of the entire adherent mucilage halo and stronger labelling at the outer periphery. In *irx14-1* seeds, CBM3a labelling was only detectable for columella and cell wall debris attached to the seed surface, consistent with the loss of mucilage adherence.

**Fig. 7.**
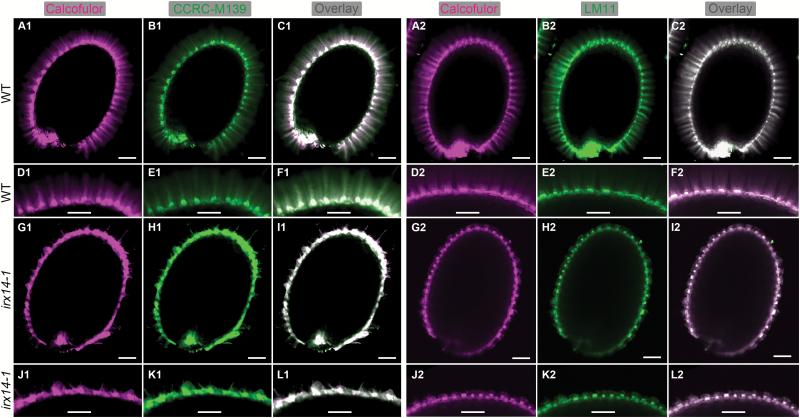
*In-situ* labelling of xylan in mucilage released from *irx14-1* and WT seeds. (A1, A2, D1, D2, G1, G2, J1, J2) Staining of β-glycans with Calcofluor White. (B1, E1, H1, K1) Detection of immunofluorescence for CCRC-M139 binding to mucilage xylan. (B2, E2, H2, K2) Immunofluorescence detection of LM11 binding to higher-substituted xylan. (C1, F1, I1, L1) Composite images of double labelling with Calcofluor and CCRC-M139. (C2, F2, I2, L2) Composite images of double labelling with Calcofluor and LM11. (D1–F1) and (J1–L1) correspond to the magnification of regions in (A1–C1) and (G1–I1), respectively. (D2–F2) and (J2–L2) are magnified regions in (A2–C2) and (G2–I2), respectively. Bar=100 μm.

Since *IRX14* was previously reported to be involved in the biosynthesis of the xylan backbone (Keppler and Showalter, 2010; Lee *et al.*, 2010; Wu *et al.*, 2010), we examined the distribution of xylan epitopes in mucilage using two xylan-specific antibodies, CCRC-M139 and LM11 (McCartney *et al.*, 2005; Pattathil *et al.*, 2010), in conjunction with Calcofluor staining (Fig. 7). The labelling of CCRC-M139 and LM11 showed similar patterns to Calcofluor in WT adherent mucilage. By comparison, CCRC-M139 and LM11 labelling was no longer observed in the adherent mucilage in *irx14-1*, and signals were only detected in the columella and remnant wall fragments attached to the seed surface. These results indicate that the distribution of cellulose and xylan in adherent mucilage in *irx14-1* is significantly disrupted.

**Fig. 8.**
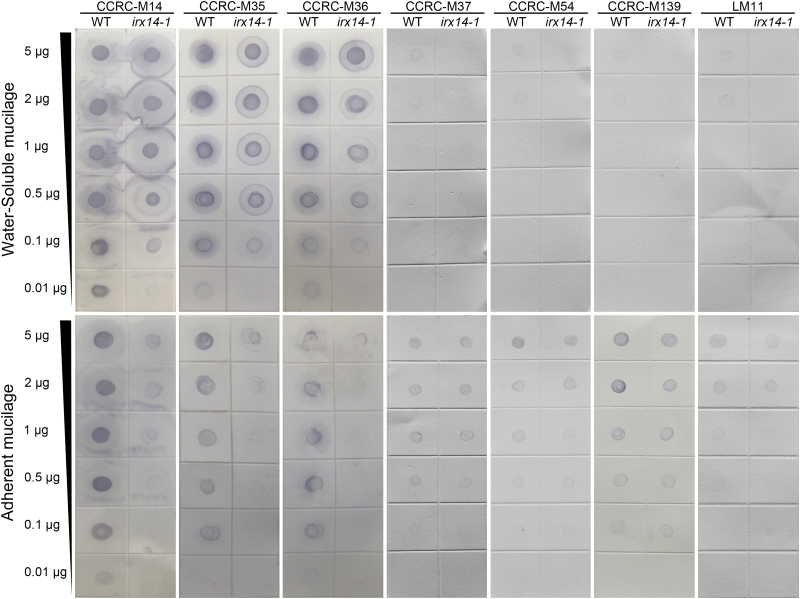
Immunoblotting of extracted mucilage of *irx14-1* and WT seeds. Water-soluble and adherent mucilage was sequentially extracted by water and 2M NaOH from *irx14-1* and WT seeds. Mucilage was diluted in a series of concentrations as specified prior to spotting on to nitrocellulose membrane. The membrane was hybridized with antibodies specifically binding to the unbranched RG I backbone (CCRC-M14, M35, and M36), and antibodies specific for xylans (CCRC-M37, M54, M139, and LM11).

To obtain more accurate information of possible changes in the structural features of xylan and RG I in *irx14-1* mucilage, dot immunoblotting was performed with three antibodies specific for the RG I backbone (CCRC-M14, CCRC-M35, and CCRC-M36) (Pattathil *et al.*, 2010) and four for xylan (CCRC-M37, CCRC-M54, CCRC-M139, and LM11) (McCartney *et al.*, 2005; Pattathil *et al.*, 2010). As shown in Fig. 8, the signal intensities of three antibodies specific for the RG I backbone were dramatically reduced in *irx14-1* adherent mucilage. In addition, the staining patterns of these three antibodies for the RG I backbone differed significantly between *irx14-1* and WT water-soluble mucilage. The labelling of CCRC-M14 in *irx14-1* water-soluble mucilage seems to be more diffuse and has a larger halo than that of the WT. As for CCRC-M35 and CCRC-M36, two distinct layers of labelling were evident for the *irx14-1* water-soluble fraction, whereas there was only a single layer of labelling for the WT. In addition, significant differences in signal intensities for xylan-specific antibodies were observed between *irx14-1* and WT mucilage. As the signal for LM10 was hardly detectable for either WT or *irx14-1* mucilage, it was excluded from the analysis. The signals of the remaining four antibodies (CCRC-M37, CCRC-M54, CCRC-M139, and LM11) were weakly detected in water-soluble mucilage in the WT, while no signals were detected in *irx14-1*. Although the epitopes of these four antibodies were detected in adherent mucilage in WT and *irx14-1*, the signal intensity was dramatically decreased in *irx14-1* compared with the WT. These results further support the conclusion that substantial structural alterations in RG I and xylan components occurred in *irx14-1* mucilage.

To obtain more detailed information of possible structural changes in *irx14-1* mucilage, the glycome-profiling of seed mucilage was analysed by utilizing cell wall glycan-directed monoclonal antibodies (Pattathil *et al.*, 2010). The mucilage of water and 2M NaOH extracts was probed using 28 monoclonal antibodies that recognize epitopes on pectin (RG-I backbone and side chains, and HG), hemicelluloses (xylan, xyloglucan, and heteromannan), and extensins. The glycome profiles of *irx14-1* mucilage displayed substantial differences compared with the WT (Supplementary Fig. S6), suggesting that *irx14-1* mucilage has undergone substantial alterations in polysaccharide structure.

## Discussion

By re-analysing the LCM seed coat microarray transcriptome data, we identified the *IRX14* gene as being involved in seed coat mucilage structure maintenance. *IRX14* has been demonstrated to be required for the elongation of the xylan backbone, and loss function of *IRX14* results in a defect in the xylan chain length and a reduction in the xylosyltransferase activity (Keppler and Showalter, 2010; Lee *et al.*, 2010; Wu *et al.*, 2010). Xylan is the major hemicellulosic polysaccharide in secondary cell walls and in grass primary cell walls. Glucuronoxylan (GX) is the major xylan in secondary cell walls of dicot plants, such as Arabidopsis and poplar. GX is composed of a linear backbone of β-(1–4)-linked d-Xyl residues, which is decorated with α-d-GlcA or 4-*O*-MeGlcA residues. In Arabidopsis seed coat mucilage, xylans represent approximately 3–5% (mol) in adherent mucilage as revealed by monosaccharide composition and glycosyl linkage analysis (Macquet *et al.*, 2007; Huang *et al.*, 2011; Walker *et al.*, 2011; Kong *et al.*, 2013; Yu *et al.*, 2014). Our results and previous studies indicated that xylan in seed mucilage had a linear backbone of β-(1–4)-linked d-Xyl with branched side chains as witnessed by the presence of 4-Xyl with a ratio of 1.8–13.3 to 2,4-Xyl (Huang *et al.*, 2011; Walker *et al.*, 2011; Kong *et al.*, 2013; Yu *et al.*, 2014; Voiniciuc *et al.*, 2015b). However, GlcA or MeGlcA was only detected in trace levels or not at all in most of these studies indicating that it is unlikely to be a side chain of xylan in seed mucilage. Although the presence of xylan as a minor component in seed mucilage has been described, its functions remain to be characterized. In this study, we show that mutations of *IRX14* reduced the xylan content in seed coat mucilage and resulted in increased detachment of adherent mucilage to the seed surface (Fig. 2). Although the *irx14-1* mutant exhibited a thinner layer of adherent mucilage, mutation of its close homologue *IRX14L* did not have any discernible effects on seed coat mucilage (Fig. 2). Similarly, mutations of *irx14* led to the reduction in xylan content and secondary wall thickness in Arabidopsis stem and the *irx14 irx14l* double mutant dramatically enhanced the phenotypes of *irx14* (Lee *et al.*, 2010; Wu *et al.*, 2010), although *irx14l* mutation did not cause any observable defect. These results also indicated that *IRX14* is functionally dominant in xylan biosynthesis in both stem and seed mucilage.

Expression analysis revealed that the transcript of *IRX14* was present throughout seed coat development with peak expression at 13 DPA (Fig. 1), corresponding to the mature green stage, at which stage mucilage synthesis completed. The expression pattern of *IRX14* was coincident with that of *CESA5* (Supplementary Fig. S1), which has been shown to be responsible for the synthesis of cellulose in seed mucilage (Sullivan *et al.*, 2011). Although cellulose microfibrils are synthesized by plasma membrane-localized cellulose synthase complexes and xylans are synthesized at the Golgi apparatus (reviewed in Driouich *et al.*, 2012), both are transported and deposited into the apoplast of the epidermal cells of the seed coat where they may form extensive cross-linking embedded in a matrix of pectic polysaccharides. Not surprisingly, mutation of the key components YIP4a, YIP4b, and ECHIDNA (ECH), which mediate the post-Golgi secretion of pectin and hemicellulose, significantly decreased the mucilage released from seeds (Gendre *et al.*, 2013). These results imply that cellulose synthesized by CESA5 and xylan synthesized by IRX14 may be simultaneously deposited into the apoplast wherein they join together to form an extensible cross-linking network.

The mucilage phenotype of *irx14-1* resembles that of several previously reported mutants affecting cellulose biosynthesis or organization in seed mucilage, such as the cellulose synthase (CESA) subunit mutant *cesa5* (Sullivan *et al.*, 2011), the leucine-rich receptor like kinases mutant *fei2* (Harpaz-Saad *et al.*, 2012), and the *COBRA-like 2* mutant *cobl2* (Ben-Tov *et al.*, 2015). Ruthenium red staining of these mutant seeds without shaking demonstrated the presence of two layers of mucilage, but only a small amount of the inner layer remained attached to the seeds after shaking (Sullivan *et al.*, 2011; Harpaz-Saad *et al.*, 2012; Ben-Tov *et al.*, 2015). The water-soluble fraction was significantly increased in *irx14-1* as observed in these mutants, which resulted in a modified distribution between the outer and the inner layers of mucilage (Fig. 4). Surprisingly, mutations in *IRX14* also led to reduced levels of crystalline cellulose in seed coat mucilage (Fig. 5; Supplementary Fig. S4). Cellulose has been shown to play important roles in anchoring the adherent mucilage to the seed coat (Sullivan *et al.*, 2011; Harpaz-Saad *et al.*, 2012). However, precisely how cellulose mediates the attachment of adherent mucilage to the seed surface remains to be clarified. Although it is possible that RG I in the adherent mucilage became entangled with cellulose microfibrils to form an extensible network, our results suggest that xylan may serve as an intermediate component to link RG I and cellulose together. This model agrees with the prevailing structural model of the cell wall that depicts the cellulose-hemicellulose network as the load-bearing component embedded in a pectin matrix (Carpita and Gibeaut, 1993). Supporting this model, the selective adsorption of xylan on to cellulose microfibrils has been extensively studied, mostly by an *in vitro* combination assay (Linder *et al.*, 2003; Kabel *et al.*, 2007; Köhnke *et al.*, 2008; Busse-Wicher *et al.*, 2014; Li *et al.*, 2015). It has been shown that xylan tends to be aligned parallel to the direction of the cellulose microfibrils, and the composition and side chain substitutions of xylan significantly affects the adsorption capacities of the cellulose fibres (Linder *et al.*, 2003; Kabel *et al.*, 2007; Busse-Wicher *et al.*, 2014). In addition, it was documented that adsorption of xylan on to cellulose-based fibres improved the physical processability of the pulp and resulted in paper with good tensile properties (Silva *et al.*, 2011). Although extensive studies indicated that xylan and cellulose could join together to form a cross-linking network, most of these studies were carried out *in vitro*, and the nature of the molecular bonding and arrangement between xylan and cellulose is still unclear. Our results indicated that xylan and cellulose might form cross-linking *in vivo* in seed coat mucilage, which is an important step toward the understanding of interactions between xylan and cellulose.

Furthermore, the reduced synthesis of xylan in seed mucilage significantly affected the macromolecular characteristics of water-soluble mucilage in *irx14-1* (Fig. 4). The larger polymeric fraction s completely disappeared in *irx14-1* and the presence of the smaller polymeric fraction was slight compared with the WT. This indicated that the polymeric fractions corresponding to aggregated or entangled polymers were structurally different in *irx14-1* mucilage. Accordingly, ELISA and the immunoblotting assay using monoclonal antibodies specific for different mucilage components (e.g. RG I, HG, xylan, etc) further confirmed that substantial alterations in ultra-structure occurred in *irx14-1* mucilage. (Fig. 8; Supplementary Fig. S6). These results indicated that cross-linking of xylan to cellulose and/or other mucilage polymers was disturbed by IRX14 mutation, and the disturbance led to further structural alterations in other components of *irx14-1* mucilage. Combined with the data presented here, we speculate that xylan may join to cellulose microfibrils and embed in the pectic polysaccharide matrix in the seed coat mucilage. Loss of xylan in *irx14-1* seed mucilage may disturb the association between xylan and cellulose and further affect the self-association of cellulose. However, how xylan cross-links with cellulose and other mucilage polymers needs to be further investigated.

The mucilage defect of *irx14-1* also resembles that of *mum5* identified in a previous forward genetic screen for modified seed mucilage (Western *et al.*, 2001), which prompted us to consider whether *IRX14* and *MUM5* are genetically identical. To test this hypothesis, the full-length coding sequence of *IRX14* was amplified by PCR using cDNA of *mum5* and sequenced. However, no point mutations were identified in the coding sequence of *IRX14* amplified from *mum5* cDNA, which indicates that *IRX14* and *MUM5* represent different alleles. The identity of *MUM5* remains unknown. Further characterization of *MUM5* function will deepen our understanding of the interaction among mucilage polymers.

In summary, the work presented here indicated that IRX14, which is involved in the biosynthesis of mucilage xylan, plays an important role in maintaining the structure of Arabidopsis seed coat mucilage. Our results demonstrated that, besides galactoglucomannan, another hemicellulose component, xylan was responsible for the structuration and adherence of the seed mucilage. These findings imply that xylans interact with cellulose to form an extensible network embedded in the pectin matrix, which is essential for anchoring the pectic adherent mucilage to the seed surface. The study expanded our understanding of the function of xylan and its interaction with cellulose, which is an important step forward in unravelling how mucilage polymers cross-link and assemble. Future studies are needed to clarify how xylan cross-links with cellulose and other mucilage components.

## Supplementary data

Supplementary data can be found at *JXB* online.

Figure S1. Heatmap showing hierarchical clustering of putative candidates and reported genes involved in mucilage biosynthesis and/or modification during seed coat development.

Figure S2. Seed coat specific expressions of four GT43 members.

Figure S3. Seed coat epidermal cell differentiation is not altered in the *irx14-1* mutant.

Figure S4. Fourier Transform Infrared Spectroscopy (FTIR) analysis of WT and *irx14-1* mucilage.

Figure S5. Water-soluble mucilage from *irx14-1* is more electrophoretically mobile than the WT.

Figure S6. Glycome profiling of the mucilage from *irx14-1* and WT seeds by ELISA assay.

Table S1. List of primers used in this study.

Supplementary Data

## References

[CIT0001] Arsovski AA Haughn GW Western TL 2010 Seed coat mucilage cells of *Arabidopsis thaliana* as a model for plant cell wall research. Plant Signaling & Behavior 5, 796–801. 20505351 10.4161/psb.5.7.11773 PMC3014532

[CIT0002] Ben-Tov D Abraham Y Stav S Thompson K Loraine A Elbaum R de Souza A Pauly M Kieber JJ Harpaz-Saad S 2015 COBRA-LIKE2, a member of the glycosylphosphatidylinositol-anchored COBRA-LIKE family, plays a role in cellulose deposition in arabidopsis seed coat mucilage secretory cells. Plant Physiology 167, 711–724. 25583925 10.1104/pp.114.240671 PMC4347734

[CIT0003] Brown DM Goubet F Wong VW Goodacre R Stephens E Dupree P Turner SR 2007 Comparison of five xylan synthesis mutants reveals new insight into the mechanisms of xylan synthesis. The Plant Journal 52, 1154–1168. 17944810 10.1111/j.1365-313X.2007.03307.x

[CIT0004] Brown DM Zhang Z Stephens E Dupree P Turner SR 2009 Characterization of IRX10 and IRX10-like reveals an essential role in glucuronoxylan biosynthesis in Arabidopsis. The Plant Journal 57, 732–746. 18980662 10.1111/j.1365-313X.2008.03729.x

[CIT0005] Busse-Wicher M Gomes TC Tryfona T Nikolovski N Stott K Grantham NJ Bolam DN Skaf MS Dupree P 2014 The pattern of xylan acetylation suggests xylan may interact with cellulose microfibrils as a twofold helical screw in the secondary plant cell wall of *Arabidopsis thaliana* . The Plant Journal 79, 492–506. 24889696 10.1111/tpj.12575 PMC4140553

[CIT0006] Carpita NC Gibeaut DM 1993 Structural models of primary cell walls in flowering plants: consistency of molecular structure with the physical properties of the walls during growth. The Plant Journal 3, 1–30. 8401598 10.1111/j.1365-313x.1993.tb00007.x

[CIT0007] Dagel DJ Liu YS Zhong L Luo Y Himmel ME Xu Q Zeng Y Ding SY Smith S 2011 In situ imaging of single carbohydrate-binding modules on cellulose microfibrils. Journal of Physical Chemistry B **115**, 635–641. 10.1021/jp109798p 21162585

[CIT0008] Dean GH Zheng H Tewari J et al 2007 The *Arabidopsis MUM2* gene encodes a β-galactosidase required for the production of seed coat mucilage with correct hydration properties. The Plant Cell 19, 4007–4021. 18165329 10.1105/tpc.107.050609 PMC2217648

[CIT0009] Driouich A Follet-Gueye ML Bernard S Kousar S Chevalier L Vicre-Gibouin M Lerouxel O 2012 Golgi-mediated synthesis and secretion of matrix polysaccharides of the primary cell wall of higher plants. Frontiers in Plant Science 3, 79. 22639665 10.3389/fpls.2012.00079 PMC3355623

[CIT0010] Ebringerová A Heinze T 2000 Xylan and xylan derivatives – biopolymers with valuable properties, 1. Naturally occurring xylans structures, isolation procedures and properties. Macromolecular Rapid Communications 21, 542–556.

[CIT0011] Francoz E Ranocha P Burlat V Dunand C 2015 *Arabidopsis* seed mucilage secretory cells: regulation and dynamics. Trends in Plant Science 20, 515–524. 25998090 10.1016/j.tplants.2015.04.008

[CIT0012] Gendre D McFarlane HE Johnson E Mouille G Sjodin A Oh J Levesque-Tremblay G Watanabe Y Samuels L Bhalerao RP 2013 *Trans*-Golgi network localized ECHIDNA/Ypt interacting protein complex is required for the secretion of cell wall polysaccharides in *Arabidopsis* . The Plant Cell 25, 2633–2646. 23832588 10.1105/tpc.113.112482 PMC3753388

[CIT0013] Gibeaut DM Carpita NC 1991 Tracing cell wall biogenesis in intact cells and plants: selective turnover and alteration of soluble and cell wall polysaccharides in grasses. Plant Physiology 97, 551–561. 16668434 10.1104/pp.97.2.551 PMC1081042

[CIT0014] Harpaz-Saad S McFarlane HE Xu S Divi UK Forward B Western TL Kieber JJ 2011 Cellulose synthesis via the FEI2 RLK/SOS5 pathway and cellulose synthase 5 is required for the structure of seed coat mucilage in Arabidopsis. The Plant Journal 68, 941–953. 21883548 10.1111/j.1365-313X.2011.04760.x

[CIT0015] Harpaz-Saad S Western TL Kieber JJ 2012 The FEI2-SOS5 pathway and CELLULOSE SYNTHASE 5 are required for cellulose biosynthesis in the Arabidopsis seed coat and affect pectin mucilage structure. Plant Signaling & Behavior 7, 285–288. 22353871 10.4161/psb.18819 PMC3405700

[CIT0016] Haughn GW Western TL 2012 *Arabidopsis* seed coat mucilage is a specialized cell wall that can be used as a model for genetic analysis of plant cell wall structure and function. Frontiers in Plant Science 3, 64. 22645594 10.3389/fpls.2012.00064 PMC3355795

[CIT0017] Huang J DeBowles D Esfandiari E Dean G Carpita NC Haughn GW 2011 The Arabidopsis transcription factor *LUH/MUM1* is required for extrusion of seed coat mucilage. Plant Physiology 156, 491–502. 21518777 10.1104/pp.111.172023 PMC3177253

[CIT0018] Kabel MA van den Borne H Vincken J-P Voragen AGJ Schols HA 2007 Structural differences of xylans affect their interaction with cellulose. Carbohydrate Polymers 69, 94–105.

[CIT0019] Kacurácová M Capek P Sasinková V Wellner N Ebringerová A 2000 FT-IR study of plant cell wall model compounds: pectic polysaccharides and hemicelluloses. Carbohydrate Polymers 43, 195–203.

[CIT0020] Keppler BD Showalter AM 2010 IRX14 and IRX14-LIKE, two glycosyl transferases involved in glucuronoxylan biosynthesis and drought tolerance in *Arabidopsis* . Molecular Plant 3, 834–841. 20595206 10.1093/mp/ssq028

[CIT0021] Köhnke T Pujolras C Roubroeks JP Gatenholm P 2008 The effect of barley husk arabinoxylan adsorption on the properties of cellulose fibres. Cellulose 15, 537–546.

[CIT0022] Kong Y Zhou G Abdeen AA et al 2013 GALACTURONOSYLTRANSFERASE-LIKE5 is involved in the production of Arabidopsis seed coat mucilage. Plant Physiology 163, 1203–1217. 24092888 10.1104/pp.113.227041 PMC3813644

[CIT0023] Laurentin A Edwards CA 2003 A microtiter modification of the anthrone-sulfuric acid colorimetric assay for glucose-based carbohydrates. Analytical Biochemistry 315, 143–145. 12672425 10.1016/s0003-2697(02)00704-2

[CIT0024] Le BH Cheng C Bui AQ 2010 Global analysis of gene activity during *Arabidopsis* seed development and identification of seed-specific transcription factors. Proceedings of the National Academy of Sciences, USA 107, 8063–8070. 10.1073/pnas.1003530107 PMC2889569 20385809

[CIT0025] Lee C Teng Q Huang W Zhong R Ye ZH 2009 The F8H glycosyltransferase is a functional paralog of FRA8 involved in glucuronoxylan biosynthesis in Arabidopsis. Plant and Cell Physiology 50, 812–827. 19224953 10.1093/pcp/pcp025

[CIT0026] Lee C Teng Q Huang W Zhong R Ye ZH 2010 The Arabidopsis family GT43 glycosyltransferases form two functionally nonredundant groups essential for the elongation of glucuronoxylan backbone. Plant Physiology 153, 526–541. 20335400 10.1104/pp.110.155309 PMC2879797

[CIT0027] Lee C Zhong R Richardson EA Himmelsbach DS McPhail BT Ye ZH 2007 The *PARVUS* gene is expressed in cells undergoing secondary wall thickening and is essential for glucuronoxylan biosynthesis. Plant and Cell Physiology 48, 1659–1672. 17991630 10.1093/pcp/pcm155

[CIT0028] Li L Perre P Frank X Mazeau K 2015 A coarse-grain force-field for xylan and its interaction with cellulose. Carbohydrate Polymers 127, 438–450. 25965503 10.1016/j.carbpol.2015.04.003

[CIT0029] Linder A Bergman R Bodin A Gatenholm P 2003 Mechanism of assembly of xylan onto cellulose surfaces. Langmuir 19, 5072–5077.

[CIT0030] Livak KJ Schmittgen TD 2001 Analysis of relative gene expression data using real-time quantitative PCR and the 2−^ΔΔ*C*^ _T_ method. Methods 25, 402–408. 11846609 10.1006/meth.2001.1262

[CIT0031] Macquet A Ralet MC Kronenberger J Marion-Poll A North HM 2007 In situ, chemical and macromolecular study of the composition of *Arabidopsis thaliana* seed coat mucilage. Plant and Cell Physiology 48, 984–999. 17540691 10.1093/pcp/pcm068

[CIT0032] McCartney L Marcus SE Knox JP 2005 Monoclonal antibodies to plant cell wall xylans and arabinoxylans. Journal of Histochemistry & Cytochemistry 53, 543–546. 15805428 10.1369/jhc.4B6578.2005

[CIT0033] Mendu V Griffiths JS Persson S Stork J Downie AB Voiniciuc C Haughn GW DeBolt S 2011 *a* Subfunctionalization of cellulose synthases in seed coat epidermal cells mediates secondary radial wall synthesis and mucilage attachment. Plant Physiology 157, 441–453. 21750228 10.1104/pp.111.179069 PMC3165890

[CIT0034] Mendu V Stork J Harris D DeBolt S 2011 *b* Cellulose synthesis in two secondary cell wall processes in a single cell type. Plant Signaling & Behavior 6, 1638–1643. 22057330 10.4161/psb.6.11.17709 PMC3329324

[CIT0035] Mortimer JC Faria-Blanc N Yu X Tryfona T Sorieul M Ng YZ Zhang Z Stott K Anders N Dupree P 2015 An unusual xylan in Arabidopsis primary cell walls is synthesised by GUX3, IRX9L, IRX10L and IRX14. The Plant Journal 83, 413–426. 26043357 10.1111/tpj.12898 PMC4528235

[CIT0036] Pattathil S Avci U Baldwin D 2010 A comprehensive toolkit of plant cell wall glycan-directed monoclonal antibodies. Plant Physiology 153, 514–525. 20363856 10.1104/pp.109.151985 PMC2879786

[CIT0037] Pena MJ Zhong R Zhou GK Richardson EA O’Neill MA Darvill AG York WS Ye ZH 2007 *Arabidopsis irregular xylem8* and *irregular xylem9*: implications for the complexity of glucuronoxylan biosynthesis. The Plant Cell 19, 549–563. 17322407 10.1105/tpc.106.049320 PMC1867335

[CIT0038] Rennie EA Scheller HV 2014 Xylan biosynthesis. Current Opinion in Biotechnology 26, 100–107. 24679265 10.1016/j.copbio.2013.11.013

[CIT0039] Scheller HV Ulvskov P 2010 Hemicelluloses. Annual Review of Plant Biology 61, 263–289. 10.1146/annurev-arplant-042809-112315 20192742

[CIT0040] Silva TCF Colodette JL Lucia LA de Oliveira RC Oliveira FN Silva LHM 2011 Adsorption of chemically modified xylans on eucalyptus pulp and its effect on the pulp physical properties. Industrial and Engineering Chemistry Research 50, 1138–1145.

[CIT0041] Stork J Harris D Griffiths J Williams B Beisson F Li-Beisson Y Mendu V Haughn G Debolt S 2010 CELLULOSE SYNTHASE9 serves a nonredundant role in secondary cell wall synthesis in Arabidopsis epidermal testa cells. Plant Physiology 153, 580–589. 20335403 10.1104/pp.110.154062 PMC2879785

[CIT0042] Sullivan S Ralet MC Berger A Diatloff E Bischoff V Gonneau M Marion-Poll A North HM 2011 CESA5 is required for the synthesis of cellulose with a role in structuring the adherent mucilage of Arabidopsis seeds. Plant Physiology 156, 1725–1739. 21705653 10.1104/pp.111.179077 PMC3149949

[CIT0043] Updegraff DM 1969 Semimicro determination of cellulose in biological materials. Analytical Biochemistry 32, 420–424. 5361396 10.1016/s0003-2697(69)80009-6

[CIT0044] Voiniciuc C Schmidt MH Berger A Yang B Ebert B Scheller HV North HM Usadel B Guenl M 2015 *a* MUCI10 produces galactoglucomannan that maintains pectin and cellulose architecture in Arabidopsis seed mucilage. Plant Physiology 169, 403–420. 26220953 10.1104/pp.15.00851 PMC4577422

[CIT0045] Voiniciuc C Yang B Schmidt MH Gunl M Usadel B 2015 *b* Starting to gel: how Arabidopsis seed coat epidermal cells produce specialized secondary cell walls. International Journal of Molecular Sciences 16, 3452–3473. 25658798 10.3390/ijms16023452 PMC4346907

[CIT0046] Walker M Tehseen M Doblin MS Pettolino FA Wilson SM Bacic A Golz JF 2011 The transcriptional regulator LEUNIG_HOMOLOG regulates mucilage release from the Arabidopsis testa. Plant Physiology 156, 46–60. 21402796 10.1104/pp.111.172692 PMC3091065

[CIT0047] Western TL 2012 The sticky tale of seed coat mucilages: production, genetics, and role in seed germination and dispersal. Seed Science Research 22, 1–25.

[CIT0048] Western TL Burn J Tan WL Skinner DJ Martin-McCaffrey L Moffatt BA Haughn GW 2001 Isolation and characterization of mutants defective in seed coat mucilage secretory cell development in Arabidopsis. Plant Physiology 127, 998–1011. 11706181 PMC129270

[CIT0049] Western TL Skinner DJ Haughn GW 2000 Differentiation of mucilage secretory cells of the Arabidopsis seed coat. Plant Physiology 122, 345–356. 10677428 10.1104/pp.122.2.345 PMC58872

[CIT0050] Willats WG McCartney L Knox JP 2001 *In-situ* analysis of pectic polysaccharides in seed mucilage and at the root surface of *Arabidopsis thaliana* . Planta 213, 37–44. 11523654 10.1007/s004250000481

[CIT0051] Windsor JB Symonds VV Mendenhall J Lloyd AM 2000 Arabidopsis seed coat development: morphological differentiation of the outer integument. The Plant Journal 22, 483–493. 10886768 10.1046/j.1365-313x.2000.00756.x

[CIT0052] Winter D Vinegar B Nahal H Ammar R Wilson GV Provart NJ 2007 An ‘Electronic Fluorescent Pictograph’ browser for exploring and analyzing large-scale biological data sets. PLoS One 2, e718. 17684564 10.1371/journal.pone.0000718 PMC1934936

[CIT0053] Wu AM Hornblad E Voxeur A Gerber L Rihouey C Lerouge P Marchant A 2010 Analysis of the Arabidopsis IRX9/IRX9-L and IRX14/IRX14-L pairs of glycosyltransferase genes reveals critical contributions to biosynthesis of the hemicellulose glucuronoxylan. Plant Physiology 153, 542–554. 20424005 10.1104/pp.110.154971 PMC2879767

[CIT0054] Wu AM Rihouey C Seveno M Hornblad E Singh SK Matsunaga T Ishii T Lerouge P Marchant A 2009 The Arabidopsis IRX10 and IRX10-LIKE glycosyltransferases are critical for glucuronoxylan biosynthesis during secondary cell wall formation. The Plant Journal 57, 718–731. 18980649 10.1111/j.1365-313X.2008.03724.x

[CIT0055] Yu L Shi D Li J Kong Y Yu Y Chai G Hu R Wang J Hahn MG Zhou G 2014 CELLULOSE SYNTHASE-LIKE A2, a glucomannan synthase, is involved in maintaining adherent mucilage structure in Arabidopsis seed. Plant Physiology 164, 1842–1856. 24569843 10.1104/pp.114.236596 PMC3982747

