# Supplementary material for: Xylan synthesized by Irregular Xylem 14 (IRX14) maintains the structure of seed coat mucilage in Arabidopsis
Source: J Exp Bot. 2016 Feb 1;67(5):1243–57. doi: 10.1093/jxb/erv510 (PMC4762376; doi:10.1093/jxb/erv510)
Supplement: Supplementary Data [file supp_67_5_1243__index.html]

Xylan synthesized by Irregular Xylem 14 (IRX14) maintains the structure of seed coat mucilage in Arabidopsis — Xylan synthesized by Irregular Xylem 14 (IRX14) maintains the structure of seed coat mucilage in Arabidopsis — Supplementary Data 

# Xylan synthesized by *Irregular Xylem 14* (*IRX14*) maintains the structure of seed coat mucilage in *Arabidopsis*

## Supplementary Data

Data files

- Supplementary\_figures\_S1\_S6\_Table\_S1.pdf - Supplementary Data
