# Supplementary material for: Xylan synthesized by Irregular Xylem 14 (IRX14) maintains the structure of seed coat mucilage in Arabidopsis
Source: J Exp Bot. 2016 Feb 1;67(5):1243–57. doi: 10.1093/jxb/erv510 (PMC4762376; doi:10.1093/jxb/erv510)
Supplement: Supplementary Data [file supp_erv510_Supplementary_figures_S1_S6_Table_S1.pdf]

Xylan synthesized by Irregular Xylem 14 (IRX14) maintains the structure of seed coat mucilage in *Arabidopsis*

Ruibo Hu, Junling Li, Xiaoyu Wang, Xun Zhao, Xuanwen Yang, Qi Tang, Guo He, , Gongke Zhou, Yingzhen Kong

Supplementary Material

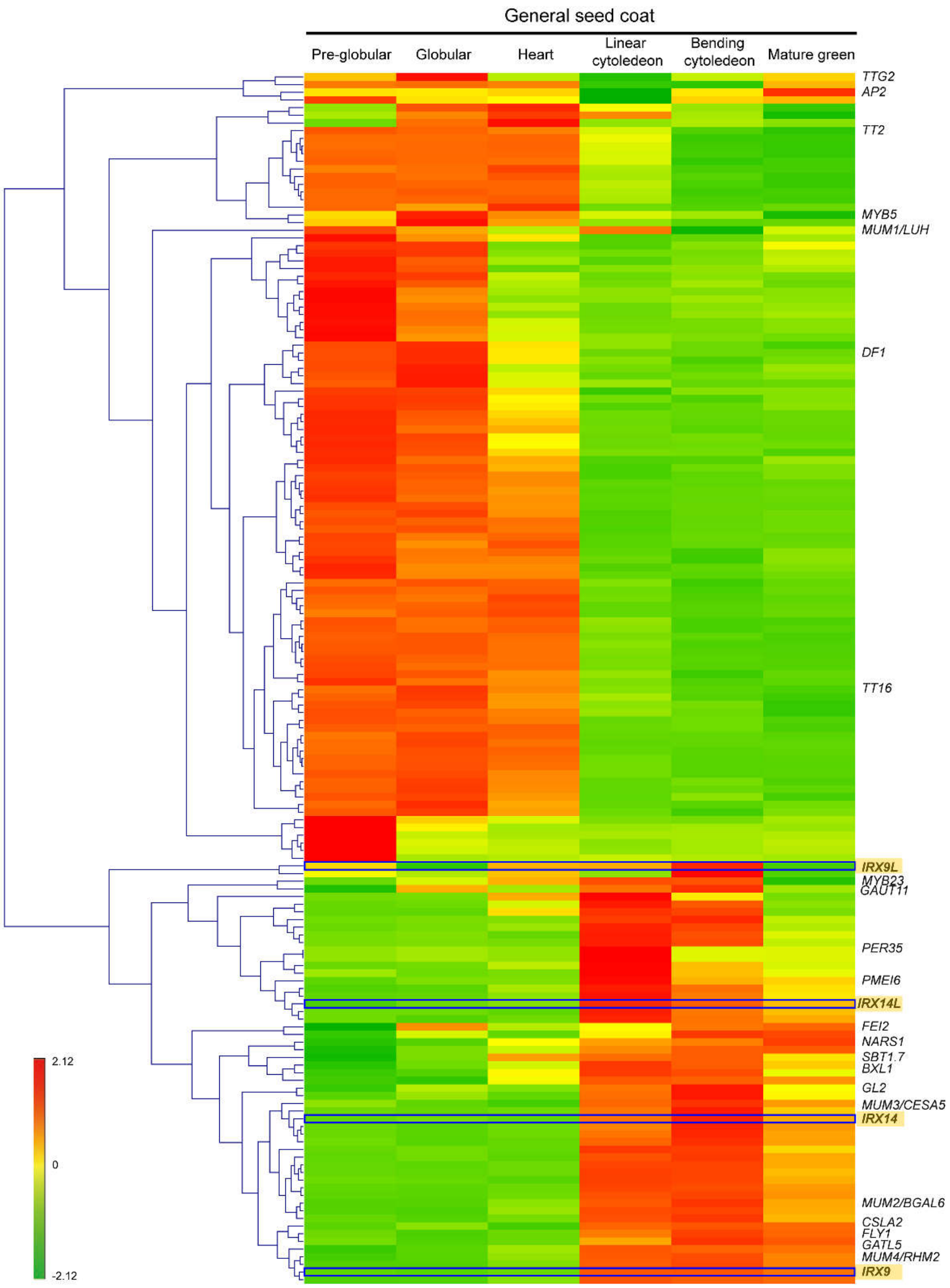

Figure S1. Heatmap showing hierarchical clustering of putative candidates and reported genes involved in mucilage biosynthesis and/or modification during seed coat development. The Affymetrix microarray data were obtained from NCBI Gene Expression Omnibus (GEO) database under the series accession number GSE12404, which consists of 42 Laser Capture Microdissection (LCM) seed samples (Le *et al.*, 2010). Four members of GT43 family (*IRX14/IRX14L*, *IRX9/IRX9L*) are highlighted. Color scale represents log2 expression values, green represents low level and red indicates high level of transcript abundances.

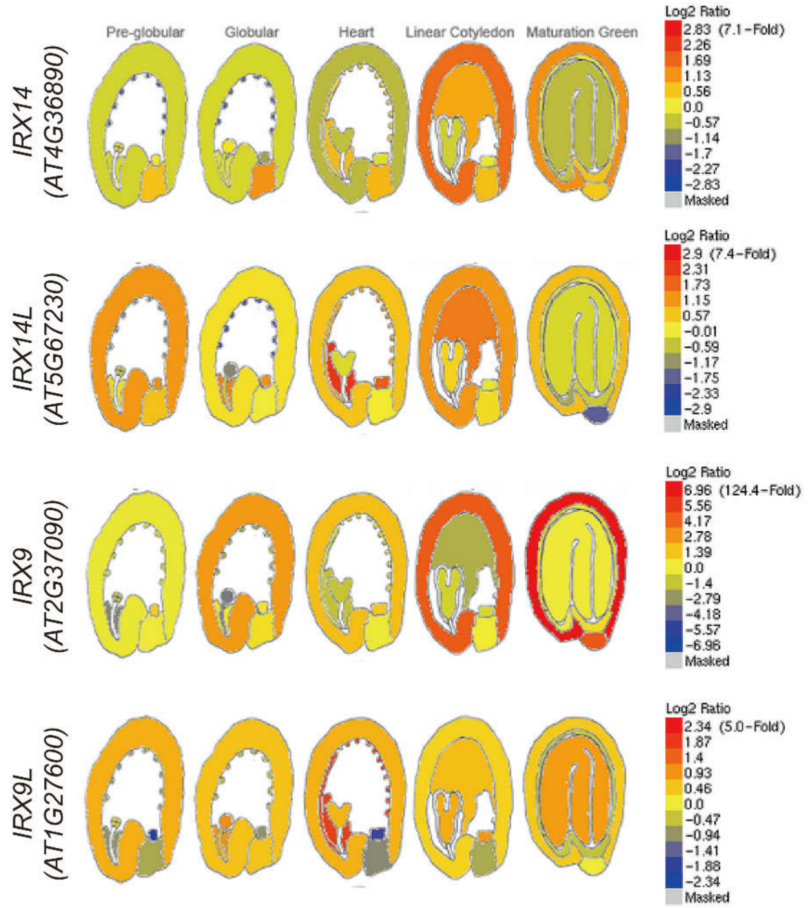

Figure S2. Seed coat specific expressions of four GT43 members.

The transcript levels of four GT43 genes (*IRX14/IRX14L*, *IRX9/IRX9L*) in the developing seed coat depicted by the BAR eFP browser (Winter *et al.*, 2007) based on the microarray data generated from LCM-dissected seed coat cells (Le *et al.*, 2010).

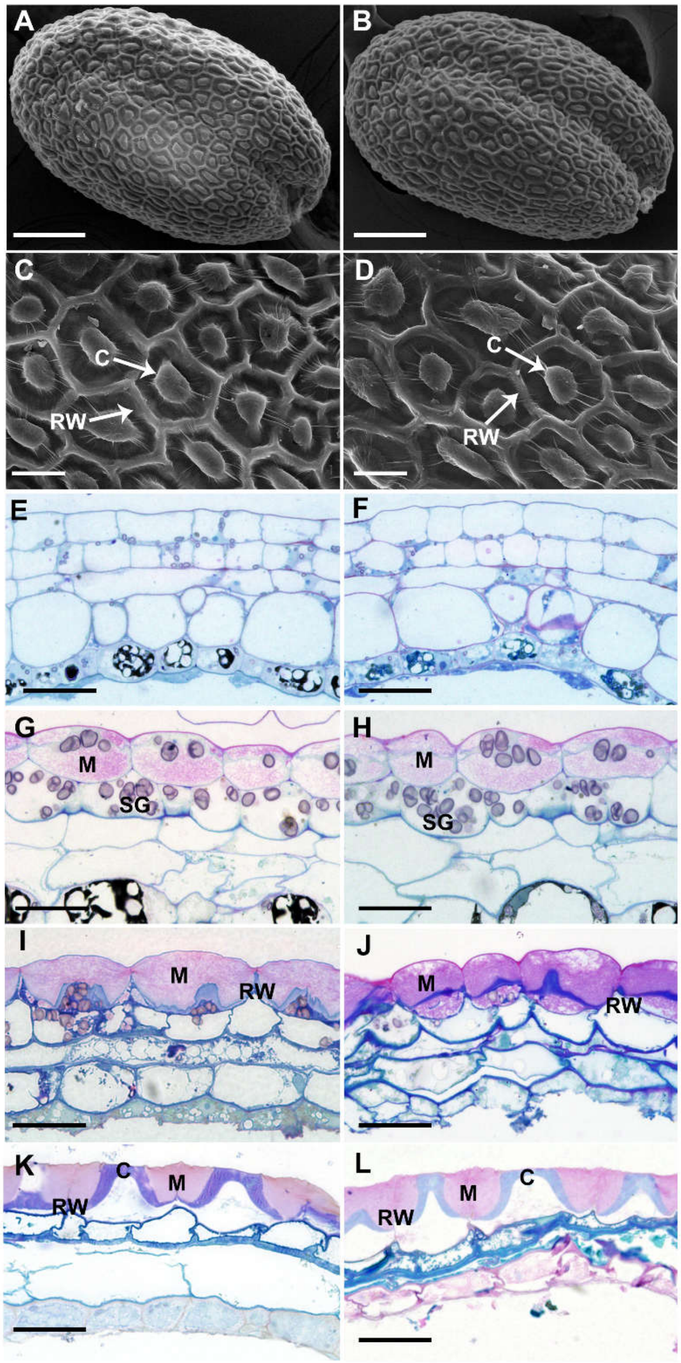

Figure S3. Seed coat epidermal cell differentiation is not altered in *irx14-1* mutant.

A to D, Scanning electron micrographs of the surface of mature dry seeds. E to L, Sections of developing seeds stained with toluidine blue O. E and F show seeds with heart stage embryos at 4 DPA. G and H show seeds with linear cotyledon stage embryos at 7 DPA. I and J show seeds with bending cotyledon stage embryos at 10 DPA. K and L show seeds with mature embryos at 13 DPA. A, C, E, G, I and K show WT seeds, and B, D, F, H, J and L show *irx14-1* seeds. C, Columella; M, mucilage; RW, radial cell wall; SG, starch granule. Bars = 100 µm (A to D) and 25 µm (C to L).

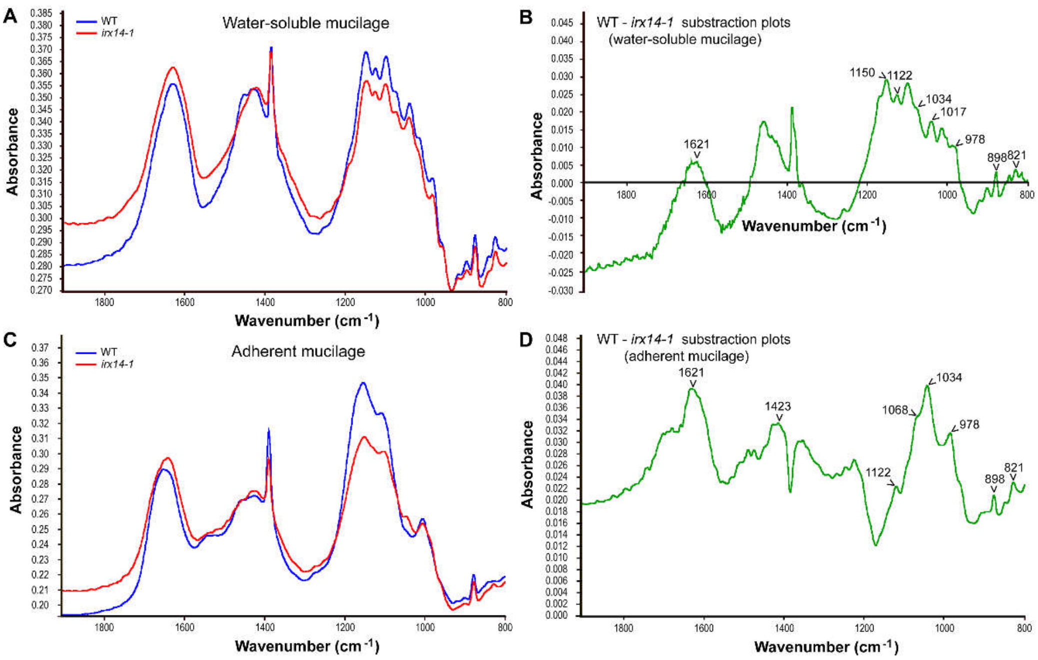

Figure S4. Fourier Transform Infrared Spectroscopy (FTIR) analysis of WT and *irx14-1* mucilage.

A, Average spectra of WT and *irx14-1* mucilage extracted with water. B, Average spectra of WT and *irx14-1* mucilage extracted with 2M NaOH. C, Digital subtraction plot of water-soluble mucilage between WT and *irx14-1* mutant. D, Subtraction plot of adherent mucilage extracted with 2 M NaOH between WT and *irx14-1*. Differences in peaks related to cellulose (896, 934, 1036 and 1065 cm^{-1}), and RGI (981, 1,019, 1,120, 1,151, 1,420 and 1,630 cm^{-1}) were labelled.

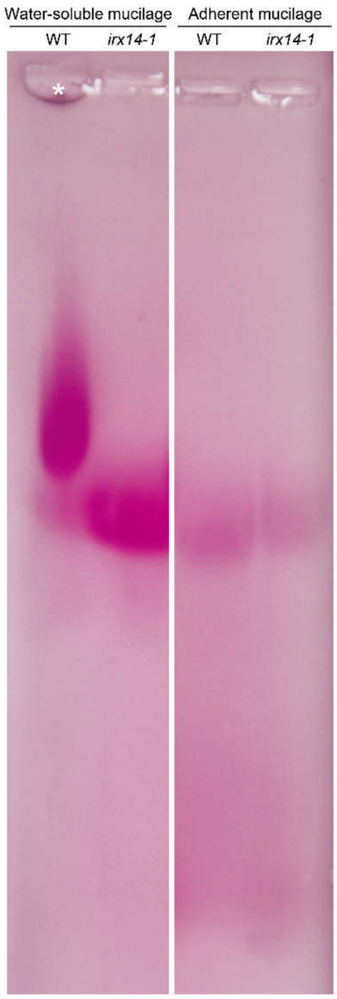

Figure S5. Water-soluble mucilage from *irx14-1* is more electrophoretically mobile than WT. Mucilage were sequentially extracted by water and 2M NaOH from WT and *irx14-1* seeds, re-suspended in water and electrophoresed in a 0.7% agarose gel. After electrophoresis, the gel were stained with Ruthenium red solution. Asterisks indicate mucilage samples remained in the loading well.

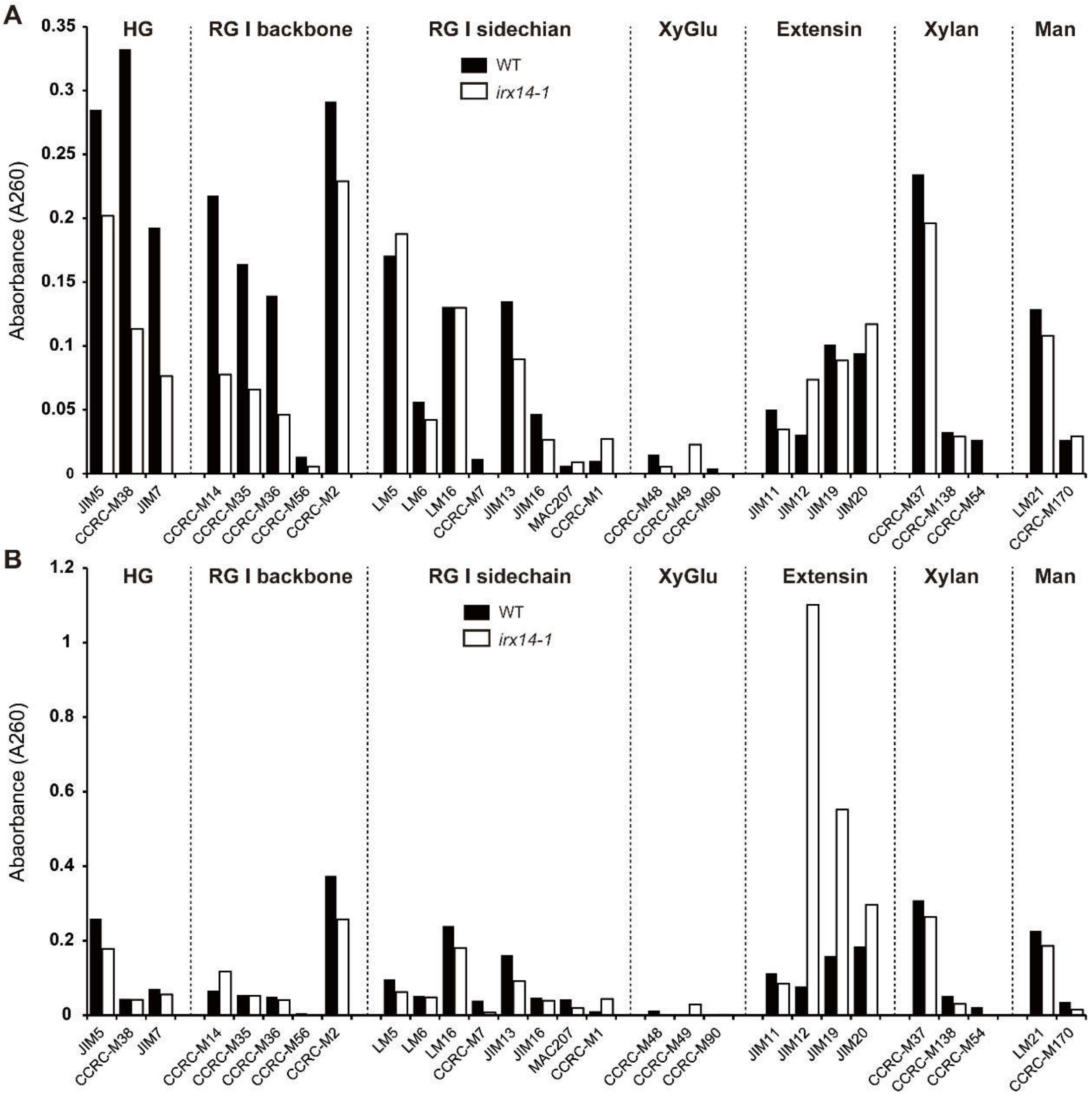

Figure. S6. Glycome profiling of the mucilage from *irx14-1* and WT seeds by ELISA assay. The mucilage of water extracts (A) and 2M NaOH extracts (B) were probed using 28 carbohydrate-directed monoclonal antibodies directed against pectin (RG-I, HG, arabinan, galactan, and arabinogalactan), hemicellulose (xylan, xyloglucan, and heteromannan), and extensin. Values represent the means of three replicates. HG, homogalacturonan; RG I, rhamnogalacturonan I; XyGlu, xyloglucan; Man, heteromannan.

Table S1. List of primers used in this study.

	Primer Name	Primer sequence (5’ to 3’)
Genotyping primers for T-DNA insertion mutants	SALK_038212_LP	AACGACACGTGTACCTCCTTG
	SALK_038212_RP	AACATCACAATCCCATCAAGC
	CS400996_LP	ATTCAGAGAAAAGGGGCAAAG
	CS400996_RP	AAAGGAGATTTGTACGGGACG
	SALK_037323_LP	CCAAAACTGTCAATTTATAACATTGG
	SALK_037323_RP	ATGTTCAATGTGCCTCAAAGC
	SALK_037323_LP	GACTAGTTGAGTCGCCTGTGC
	SALK_037323_RP	CTTCCATCTCGCTTTCATCAG
	SALK_066961_LP	CTTGCTCTTCGACACTCTTGG
	SALK_066961_RP	ATCGATGTACGGTGTGAGGAG
qRT-PCR primers	*AtIRX14*_214F	ACCAATCTCTACTCTCTTCCGTTTC
	*AtIRX14*_321R	CACCACCGTCGTTCCGTTAG
	*AtACT5*_222F	AAGACGGATGATTGTAGG
	*AtACT5*_324R	CATATAATAAGGCTCCAAGAAT
In situ hybridization	*AtIRX14*_Pb_146F	TGATTAGTCTAGTTCTCGGCTTTCG
	*AtIRX14*_Pb_321R	CACCACCGTCGTTCCGTTAG
Gene cloning	*AtIRX14*_1F	ATGAAGCTCTCTGCTTTACATCA
	*AtIRX14*_1578R	TCAGTTTCTTTCTTGATGCTTAGAC
